# Phenome-Wide Association Studies on a Quantitative Trait: Application to TPMT Enzyme Activity and Thiopurine Therapy in Pharmacogenomics

**DOI:** 10.1371/journal.pcbi.1003405

**Published:** 2013-12-26

**Authors:** Antoine Neuraz, Laurent Chouchana, Georgia Malamut, Christine Le Beller, Denis Roche, Philippe Beaune, Patrice Degoulet, Anita Burgun, Marie-Anne Loriot, Paul Avillach

**Affiliations:** 1 Biomedical Informatics and Public Health Department, University Hospital HEGP, AP-HP, Paris, France; 2 INSERM UMR_S 872 Team 22: Information Sciences to support Personalized Medicine, Université Paris Descartes, Sorbonne Paris Cité, Faculté de Médecine, Paris, France; 3 INSERM UMR-S 775, Université Paris Descartes, Sorbonne Paris Cité, Paris, France; 4 Gastroenterology Department, University Hospital HEGP, AP-HP, Paris, France; 5 Pharmacovigilance Center, University Hospital HEGP, AP-HP, Paris, France; 6 Biochemistry, Pharmacogenetics and Molecular Oncology Unit, University Hospital HEGP, AP-HP, Paris, France; Tufts University, United States of America

## Abstract

Phenome-Wide Association Studies (PheWAS) investigate whether genetic polymorphisms associated with a phenotype are also associated with other diagnoses. In this study, we have developed new methods to perform a PheWAS based on ICD-10 codes and biological test results, and to use a quantitative trait as the selection criterion. We tested our approach on thiopurine *S*-methyltransferase (TPMT) activity in patients treated by thiopurine drugs. We developed 2 aggregation methods for the ICD-10 codes: an ICD-10 hierarchy and a mapping to existing ICD-9-CM based PheWAS codes. Eleven biological test results were also analyzed using discretization algorithms. We applied these methods in patients having a TPMT activity assessment from the clinical data warehouse of a French academic hospital between January 2000 and July 2013. Data after initiation of thiopurine treatment were analyzed and patient groups were compared according to their TPMT activity level. A total of 442 patient records were analyzed representing 10,252 ICD-10 codes and 72,711 biological test results. The results from the ICD-9-CM based PheWAS codes and ICD-10 hierarchy codes were concordant. Cross-validation with the biological test results allowed us to validate the ICD phenotypes. Iron-deficiency anemia and diabetes mellitus were associated with a very high TPMT activity (p = 0.0004 and p = 0.0015, respectively). We describe here an original method to perform PheWAS on a quantitative trait using both ICD-10 diagnosis codes and biological test results to identify associated phenotypes. In the field of pharmacogenomics, PheWAS allow for the identification of new subgroups of patients who require personalized clinical and therapeutic management.

## Author Summary

The use of underlying molecular mechanisms and other factors to describe and classify diseases is a major challenge for future treatment strategies. New methods are needed to achieve this goal. The phenome wide association study (PheWAS) methodology was initially developed to unveil unknown associations between a specific genetic status and phenotypic features (e.g. diagnoses from electronic health records). We initially propose to extend this method to assessment of the relationships between the levels of a quantitative trait and diagnosis codes. We also assess the relationships between this quantitative trait and the biological test results. We tested this method using the levels of enzymatic activity of thiopurine *S*-methyltransferase (TPMT) that is involved in the metabolism of thiopurine drugs used in inflammatory bowel diseases for example. We discovered an association between a very high TPMT activity and nutritional anemia and diabetes. These results could be used to describe a new subgroup of patients in order to optimize drug treatments.

## Introduction

The US National Research Council report “Toward Precision Medicine” proposed the redefinition of diseases using the underlying molecular causes and other factors in addition to traditional signs and symptoms [1]. To establish the relationships between molecular characterization and clinical features, different methods have been proposed [2]. Genome Wide Association Studies (GWAS) have allowed the identification of Single Nucleotide Polymorphisms (SNPs) associated with a determinate phenotype [3]–[5]. (Figure S1 Panel A) Between 2005 and June 2012, 1,350 GWAS were published [6]. In 2010, Denny *et al.* described another method called Phenome-Wide Association Study (PheWAS) [7]. PheWAS investigates whether the SNPs associated with a phenotype are also associated with other diagnoses (Figure S1 Panel B) [7], [8]. Therefore, for a selected SNP, two groups are composed: one with a specific allele and a control group with other alleles. Thereafter, to search for new associations, all of the phenotypic data (for example, all International Classification of Diseases (ICD) codes) available in the medical records of the patients having the specific allele are screened and compared to those of the control group [9]. Denny *et al.* genotyped 6,000 patients in the BioVU data bank at five SNPs with previously reported disease associations and ran a PheWAS on each SNP, based on the ICD-9-CM codes [7], [10]. They replicated four out of seven known molecular-clinical associations and discovered 19 new potential associations.

Following this example, further PheWAS were performed on the SNPs associated with hypothyroidism (*FOXE1*) [8], rheumatoid arthritis [11], and on HLA-DRB1*1501, which has been linked to several autoimmune diseases [12]. Most PheWAS were performed with data collected through the Electronic Medical Records and Genomics (eMERGE) network, including the Marshfield Clinic's Personalized Medicine cohort [13]. With the aim of analyzing the genetic architecture of complex traits and identifying new pleiotropic relationships, Pendergrass *et al.* conducted a PheWAS on 70,061 study participants representing four major racial/ethnic groups in the Population Architecture using Genomics and Epidemiology (PAGE) network [14], [15].

Analyses combining GWAS and PheWAS have been reported: whereas GWAS allows researchers to identify a genomic region of interest or one SNP associated with a clinical condition, PheWAS identifies all the diagnoses potentially associated with these markers. For example, Denny *et al.* performed a GWAS for primary hypothyroidism and, afterwards a PheWAS on 13,617 patient records, based on the locus that was previously identified. Thus, genetic associations with thyroiditis and thyrotoxicosis but neither Graves or thyroid cancer have been highlighted [8]. More recently, Ritchie *et al.* performed genome- and phenome-wide analysis on cardiac conduction, which resulted in the identification of new markers for atrial fibrillation and arrhythmia [16].

To perform a PheWAS, a large amount of data must be included to infer potential patterns and discover new possible associations [17], [18]. The criterion for data selection includes the presence of a particular genotype. A cohort containing all types of diagnoses is necessary to discover some new potential associations. Clinical Data Warehouses (CDWs) have been developed to allow the integration of Electronic Health Records (EHRs) data and their use for research; they can also be used as data source for such studies [19]–[22]. When linked to DNA repositories, CDWs are a source of patient data to analyze the relationship between genetic variations and human traits [23]–[26].

Instead of directly using genomic data as the inclusion criteria, it is possible to use a quantitative trait (*e.g.*, biological test results) [27]. This approach presents three advantages: (i) quantitative traits are usually recorded as part of the clinical data; (ii) a quantitative trait, consisting of both genetic variations and non-genetic factors can more accurately describe a clinical feature than genetic mutations alone; (iii) quantitative traits can be highly correlated to a genomic status. This is the case for thiopurine *S*-methyltransferase (TPMT), a key enzyme involved in thiopurine metabolism, as TPMT activity is highly correlated to the genotypes of individuals [28]–[30].

Thiopurine drugs (azathioprine, 6-thioguanine and 6-mercaptopurine) are frequently prescribed in autoimmune disorders, such as inflammatory bowel disease (IBD), or in blood cancers, such as acute lymphoblastic leukemia [29], [31]. Severe adverse effects occur in 15% to 28% of the treated patients, and up to 40% of IBD patients are resistant to thiopurines [29], [32], [33]. The production of active metabolites, such as the 6-thioguanine nucleotides (6-TGN), is largely regulated by TPMT [33], [34]. Genetic polymorphisms of TPMT result in a trimodal distribution of TPMT activity (TPMTa). Whereas a large majority, approximately 89%, of the population show normal activity (nTPMTa), approximately 11% have a partially deficient activity level, and 0.3% have a completely deficient activity level [30], [35], [36]. Moreover, among patients with nTPMTa, approximately 15% show a very high TPMTa (vhTPMTa) [29], [37].

In treated patients, there is a negative correlation between partial or completely deficient TPMTa, and high 6-TGN intra-erythrocyte concentrations, resulting in severe hematological toxicities or even lethal bone marrow suppression [34]. Conversely, patients with vhTPMTa are more prone to low 6-TGN intra-erythrocyte concentrations and pharmacological resistance to thiopurines [38]. Therefore, to detect patients at high risk of severe hematological toxicities, the US Food and Drug Administration (FDA) and the Clinical Pharmacogenetics Implementation Consortium (CPIC) strongly recommend that TPMT status be determined either by genotyping or phenotyping prior to initiation of thiopurine therapy [30]. Based on these observations, TPMTa levels can be used as a starting point for a PheWAS.

### Objectives

We aimed to develop methods to perform a PheWAS based on the ICD-10 codes and biological test results, while using a quantitative trait as a selection criterion. We then tested our approach on a specific quantitative trait, TPMTa, in order to identify new subgroups of patients with different characteristics.

## Materials and Methods

### Study and clinical data warehouse

We performed an *in silico* retrospective case-control study using data from an academic hospital, Hôpital Européen Georges Pompidou (HEGP) in Paris, France. We extracted data from HEGP CDW, an i2b2 CDW containing more than 606,524 single patients, collected between 2000 and 2012 [20], [39]. This CDW contains routine care data divided into nine categories (208,955,369 items): demographics (age, sex, and hospital vital status), vital signs (*e.g.*, temperature, blood pressure, weight…), diagnoses (ICD-10), procedures (French CCAM classification), clinical data (structured questionnaires from EHR), free text reports, pathology codes (French ADICAP classification), biological test results, and Computerized Provider Order Entry (CPOE) drug prescriptions.

### Definition of ICD-10 PheWAS codes: Two aggregation levels

ICD codes could not be directly used for analysis because of their fine granularity. Therefore, we developed two different aggregation methods.

#### ICD-9-CM mapping PheWAS codes

**Figure 1:**
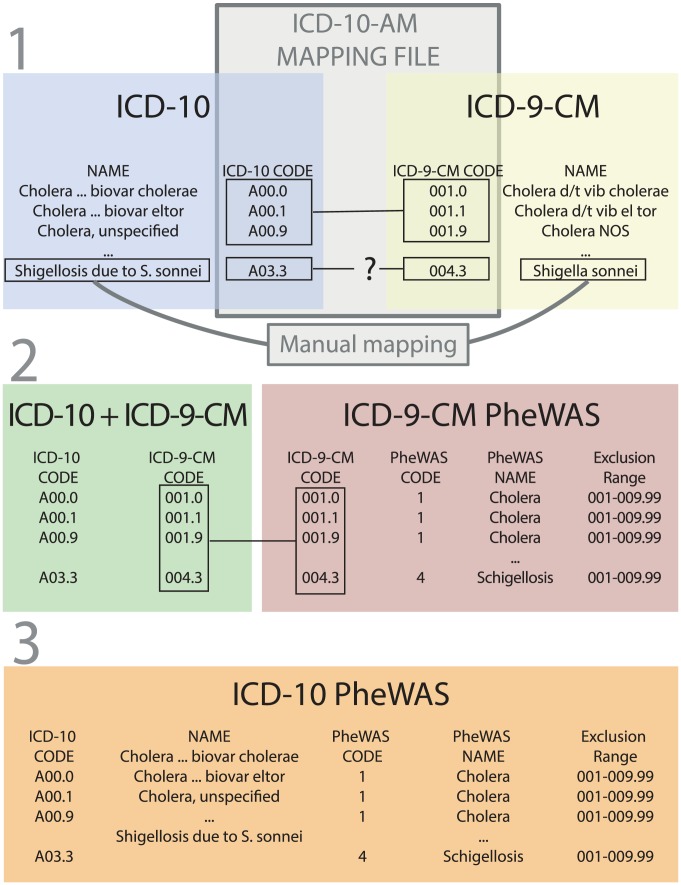
Three steps terminology construction, from PheWAS codes based on ICD-9-CM to PheWAS codes based on ICD-10 using ICD-9-CM-A to ICD-10-AM mapping file and manual mapping. PheWAS: Phenome-wide association study; ICD: International classification of diseases; ICD-9-CM: International classification of diseases clinically modified; ICD-9-CM-A: Australian version of the ICD-9-CM, with custom codes added. ICD-10-AM: Australian version of the ICD-10, with custom codes added. 1: Mapping file from the New-Zealand Ministry of Health was used to project ICD-10 codes on ICD-9-CM. 2: Mapping of the previous projection with existing ICD-9-CM PheWAS codes. 3: File with correspondence between ICD-10 codes and ICD-9-CM PheWAS codes.

The first aggregation scale relies on mapping between ICD-9-CM and ICD-10 [40]. We extracted the ICD-10 classification from the United Medical Language System (UMLS) [41]. Then, we used a mapping file developed by the New Zealand Ministry of Health to map the ICD-10 codes to the ICD-9-CM codes (Figure 1) [40]. After format adaptations, 99.5% of the codes were mapped successfully. The 57 remaining codes were mapped manually. This allowed us to use the ICD-9-CM PheWAS codes from Denny *et al.* [42] These ICD-9-CM PheWAS codes contained 829 different codes including 771 used for analysis. Codes that were not a proper diagnosis were excluded (*e.g.* “Effects of air pressure caused by explosion”).

#### ICD-10 PheWAS codes

The other grouping method was based on the ICD-10 hierarchy. Given the size of our sample population, a lower level of granularity was more relevant. We used the superclasses of the three digit codes, leading to 257 groups. ICD-10PheWAS codes and ICD-10 to ICD-9-CM PheWAS codes mapping files are available for download here: http://umrs872eq22.com/TPMT_PLOS/Phewas_codes_ICD10_ICD9.zip

### ICD codes analyses

**Figure 2:**
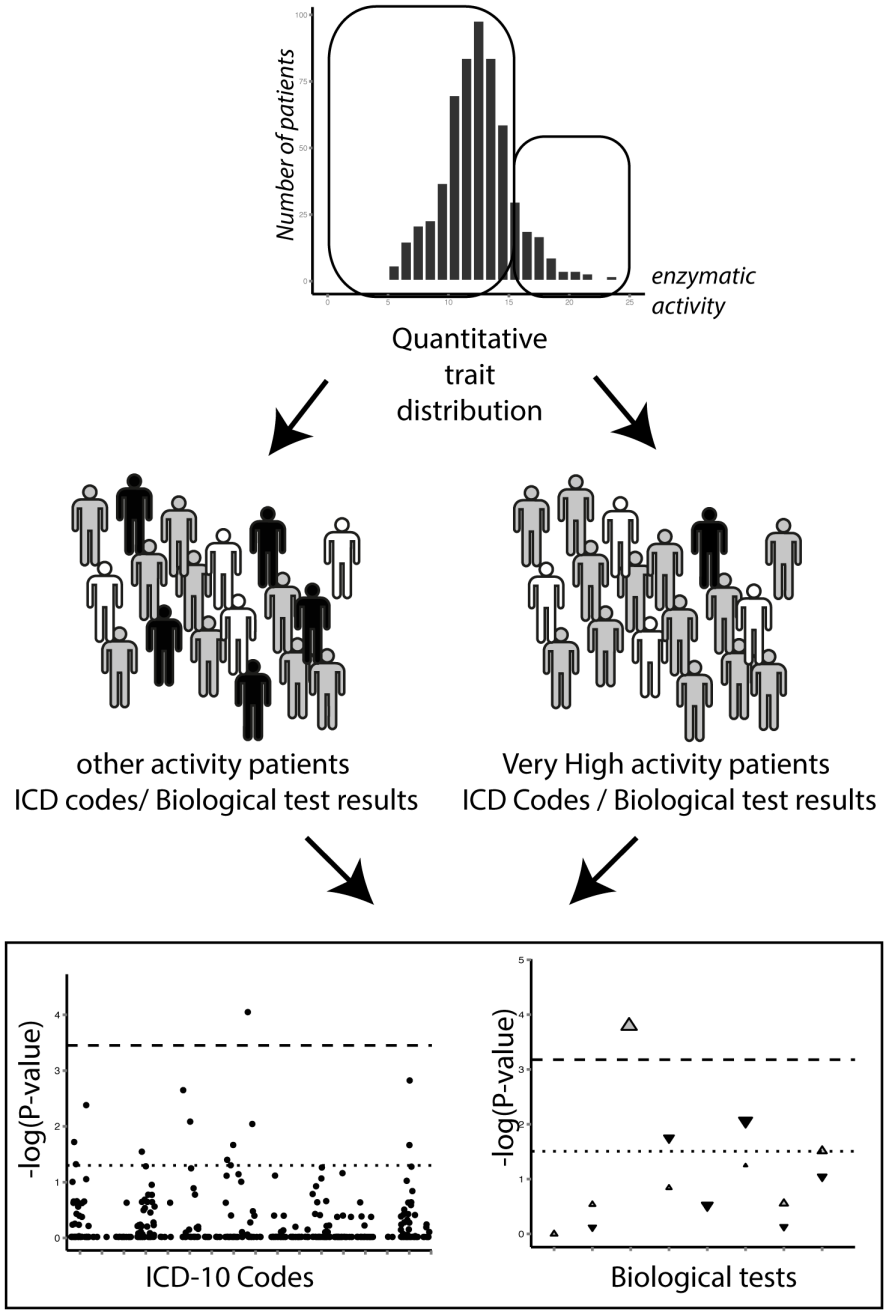
Schematic representation of a PheWAS on a quantitative trait, analyzing ICD codes and biological test results. PheWAS: Phenome-wide association study; ICD: International classification of diseases; TPMT: thiopurine *S*-methyltransferase. Patients are assigned to a group depending on the level of a quantitative trait (*e.g.* TPMT activity). ICD codes and biological test results are screened to find systematic differences between the groups.

Groups of patients were divided according to the quantitative trait studied. (Figure 2) Then, as described by Denny *et al.*, for each PheWAS code, a case-control comparison was performed: (i) the case group was generated with patients having an ICD code in the range of this PheWAS code; (ii) the control group was composed of patients without any ICD code in this range; and (iii) patients with ICD codes that were too close to those of the current PheWAS code were excluded from this specific comparison. For each PheWAS code, its siblings were used as exclusion ranges. For example: for grouped codes under C15–26 (Malignant neoplasms, digestive organs), the exclusion range was from C00 to D48 (Neoplasms). We successively used the two methods of ICD code aggregation to compare the distribution of cases between the groups.

### Biological test result analyses

For each biological test, we used thresholds to define low-value cases and high-value cases, according to the normal value range (Table 1). Because patients had more than one occurrence of each biological test, two algorithms of analyses were applied. The first was a “global approach” in which a high-value case (resp. a low-value case) was defined as the presence of at least one test result above the high threshold (resp. below the low threshold). (Figure S2) In addition, for hyperglycemia, we required two occurrences above the high threshold. (Table S1) The proportions of cases among patient groups were then compared, similar to the ICD analysis. The second method was a “frequency based approach” in which a case was defined by one encounter with at least one result either below (low-value cases) or above (high-value cases) the thresholds. (Figure S2) The proportions of encounter cases (“episodes”) per patient for a test were compared, similar to the ICD analysis. The results of the “global approach” and of the “frequency-based approach” were analyzed in view of the ICD-based findings.

**Table 1:** Description of electronic health records (EHRs) of the thiopurine S-methyltransferase activity tested patients (TPMT cohort) and the control patients.

		*TPMT cohort*				Control Patients (%) n = 1668
		Low TPMTa (%) n = 52	Normal TPMTa (%) n = 413	Very high TPMTa (%) n = 89	All (%) n = 554	
Year of birth	1900–1910	0 (0)	0 (0)	0 (0)	0 (0)	0 (0)
	1911–1920	0 (0)	4 (1)	1 (1.1)	5 (0.9)	12 (0.7)
	1921–1930	1 (1.9)	11 (2.7)	1 (1.1)	13 (2.3)	42 (2.5)
	1931–1940	4 (7.7)	33 (8)	6 (6.7)	43 (7.8)	120 (7.2)
	1941–1950	6 (11.5)	36 (8.7)	8 (9)	50 (9)	147 (8.8)
	1951–1960	6 (11.5)	51 (12.3)	14 (15.7)	71 (12.8)	216 (12.9)
	1961–1970	9 (17.3)	81 (19.6)	18 (20.2)	108 (19.5)	321 (19.2)
	1971–1980	11 (21.2)	90 (21.8)	20 (22.5)	121 (21.8)	372 (22.3)
	1981–1990	13 (25)	91 (22)	16 (18)	120 (21.7)	369 (22.1)
	1991–2000	2 (3.8)	16 (3.9)	5 (5.6)	23 (4.2)	69 (4.1)
	2001–2010	0 (0)	0 (0)	0 (0)	0 (0)	0 (0)
Sex	Male	33 (63.5)	202 (48.9)	40 (44.9)	275 (49.6)	843(50.5)
	Female	19 (36.5)	211 (51.1)	49 (55.1)	279 (50.4)	825(49.5)
Unit	Hepato-gastro enterology	-	-	-	2,667 (60.7)	393 (9.2)
	Digestive surgery	-	-	-	292 (6.6)	275 (6.4)
	Internal medicine	-	-	-	303 (6.9)	245 (5.7)
	Nephrology	-	-	-	272 (6.2)	237 (5.6)
	Pneumology	-	-	-	220 (5)	177 (4.1)
	ER	-	-	-	169 (3.8)	242 (5.7)
	Cardiovascular surgery	-	-	-	84 (1.9)	343 (8)
	Vascular medicine	-	-	-	82 (1.9)	320 (7.5)
	Cardiology	-	-	-	80 (1.8)	253 (5.9)
	Radiotherapy	-	-	-	36 (0.8)	133 (3.1)
	Immunology	-	-	-	33 (0.8)	153 (3.6)
	Anesthesia - Surgical intensive care	-	-	-	29 (0.7)	90 (2.1)
	Thoracic surgery	-	-	-	22 (0.5)	104 (2.4)
	Medical intensive care	-	-	-	23 (0.5)	36 (0.8)
	Otolaryngology	-	-	-	19 (0.4)	201 (4.7)
	Gynaecologic surgery	-	-	-	16 (0.4)	226 (5.3)
	Orthopedics	-	-	-	13 (0.3)	323 (7.6)
	Ambulatory surgery	-	-	-	13 (0.3)	147 (3.4)
	Preventive cardiovascular medicine	-	-	-	8 (0.2)	91 (2.1)
	Urology	-	-	-	7 (0.2)	49 (1.1)
	Cardiovascular radiology	-	-	-	4 (0.1)	12 (0.3)
	Plastic surgery	-	-	-	0 (0)	15 (0.4)
	Oncology	-	-	-	0 (0)	205 (4.8)
Encounter Type	Hospitalization	179 (46.3)	1,123 (38.4)	246 (42.8)	1,594 (39.7)	2,010 (34.2)
	Consultation	53 (13.7)	487 (16.7)	95 (16.5)	646 (16.1)	1,549 (26.4)
	Others	1 (0.3)	0 (0)	0 (0)	2 (0)	135 (2.3)
	Post acute care	1 (0.3)	5 (0.2)	0 (0)	7 (0.2)	18 (0.3)
	Session	83 (21.4)	825 (28.2)	158 (27.5)	1,118 (27.9)	1,000 (17)
	Emergency Unit	70 (18.1)	484 (16.6)	76 (13.2)	644 (16.1)	1,163 (19.8)

Control patients are randomly extracted from the clinical data warehouse (CDW) of the Hôpital européen Georges Pompidou.

TPMTa: thiopurine *S*-methyltransferase activity; low TPMTa: <8.5 nmol/h/mL red blood cells; very high TPMTa: ≥15.0 nmol/h/mL red blood cells; normal TPMTa: in between.

For biological tests significantly associated to a TPMTa group, we performed an event-free Kaplan-Meier survival analysis (*i.e.* low-value event or high-value event) after initiation of thiopurine therapy, excluding events occurring within the first week of treatment. Analysis was censored to 360 days after initiation of thiopurine therapy.

### Application to TPMT enzyme activity

#### Population

We selected all the patients who underwent a TPMTa assay and with at least one ICD-10 code or one biological test result between January 2000 and July 2013, *i.e. TPMT cohort*. For the PheWAS analysis, we included the patients having a notion of thiopurine treatment in their EHR and kept ICD codes and biological test results dated after the starting of thiopurine treatment. There were no exclusion criteria. We will refer to this group as the *study population*.

We first compared the characteristics of the *TPMT cohort* to a hospital control group composed of randomly selected patients among the HEGP CDW who did not undergo a TPMTa assessment and were matched for year of birth and sex (3 for each patient in the *TPMT cohort*).

Then, we split the initial *TPMT cohort* into three groups according to TPMTa level: (i) low TPMTa (lowTPMTa) combining both partial and completely deficient TPMTa patients, with an activity below 8.5 nmol/h/mL red blood cells (RBC); (ii) nTPMTa; and (iii) vhTPMTa, with an activity above 15.0 nmol/h/mL RBC [43]. We have assessed that TPMTa is stable over time from the patients (n = 51) who underwent more than one TPMTa assay (Table S2). For these patients, only the first measurement was used in the analyses.

#### Data management

An open database connection (ODBC) linking an Oracle database (11 g Enterprise Edition Release 11.2.0.1.0) of i2b2 CDW (version 1.3) to R software (version 2.15.3) was set up. The dataset containing data from the *TPMT cohort* (demographic, diagnoses, free text reports, structured questionnaires, biological tests results and drug prescriptions) was imported into R. All further analyses were carried out in R, using the RODBC 1.3–6 and the ggplot2 0.9.3.1 packages.

#### Time restrictions

The information concerning the treatment was found in the drug prescriptions, in free text reports or in clinical data from structured questionnaires. We extracted prescriptions from the CPOE drug prescriptions with starting dates or directly from free text reports using the brand name and the generic name (IMUREL, AZATHIOPRINE, IMURAN, MERCAPTOPURINE, PURINETHOL) and using the date of report as the starting date.

#### ICD codes analysis

We compared the proportions of cases and controls in the TPMTa groups: (i) vhTPMTa versus other TPMTa and the (ii) lowTPMTa versus other TPMTa. We selected the PheWAS codes with at least 5 occurrences for analysis.

#### Biological test result analyses

Among the biological tests, we focused on 11 routine blood tests widely prescribed during the monitoring of thiopurine treatment: leukocyte count (WBC), neutrophil count, RBC count, hemoglobin, platelet count, mean corpuscular volume (MCV), glycemia, alkaline phosphatase (ALP), alanine aminotransferase (ALT), aspartate aminotransferase (AST), and gamma glutamyl-transpeptidase (GGT) (Table S1).

#### Thiopurine efficacy analysis on free-text reports

From study population, we selected the patients having at least two free-text reports with a reference to thiopurine therapy in their EHR. We excluded the patients with a reported adverse effect or intolerance to azathioprine or 6-mercaptopurine, and the patients whose treatment was interrupted within the first month.

Thiopurine failure was defined as at least one reference to inefficiency/failure of azathioprine/6-mercaptopurine therapy, or as a sustained dependency to steroids, reported by physicians in free-text reports. Of note, if the treatment was initially reported as effective, a secondary failure was not considered in our analysis. Proportions of thiopurine therapy failure were compared between vhTPMTa patients and other TPMTa patients.

### Statistical analyses

Fisher exact test and unadjusted logistic regression were used to compare discrete variables. Continuous variables were compared using Student t-test. Log-rank test was used to evaluate survival curves. We calculated the odds ratios (OR) and confidence intervals at 95% (95%CI). q-q plots were realized to evaluate the distribution of p-values. The p-value was fixed at 0.05. We used the False Discovery Rate (FDR) method to manage multiple testing and used the threshold of 0.2 [44].

### Ethics statement

This study was approved by the IRB and ethics committee CPP Ile-de-France II. IRB Committee # 00001072. Study reference # CDW_2013_0002.

## Results

### Participants

**Figure 3:**
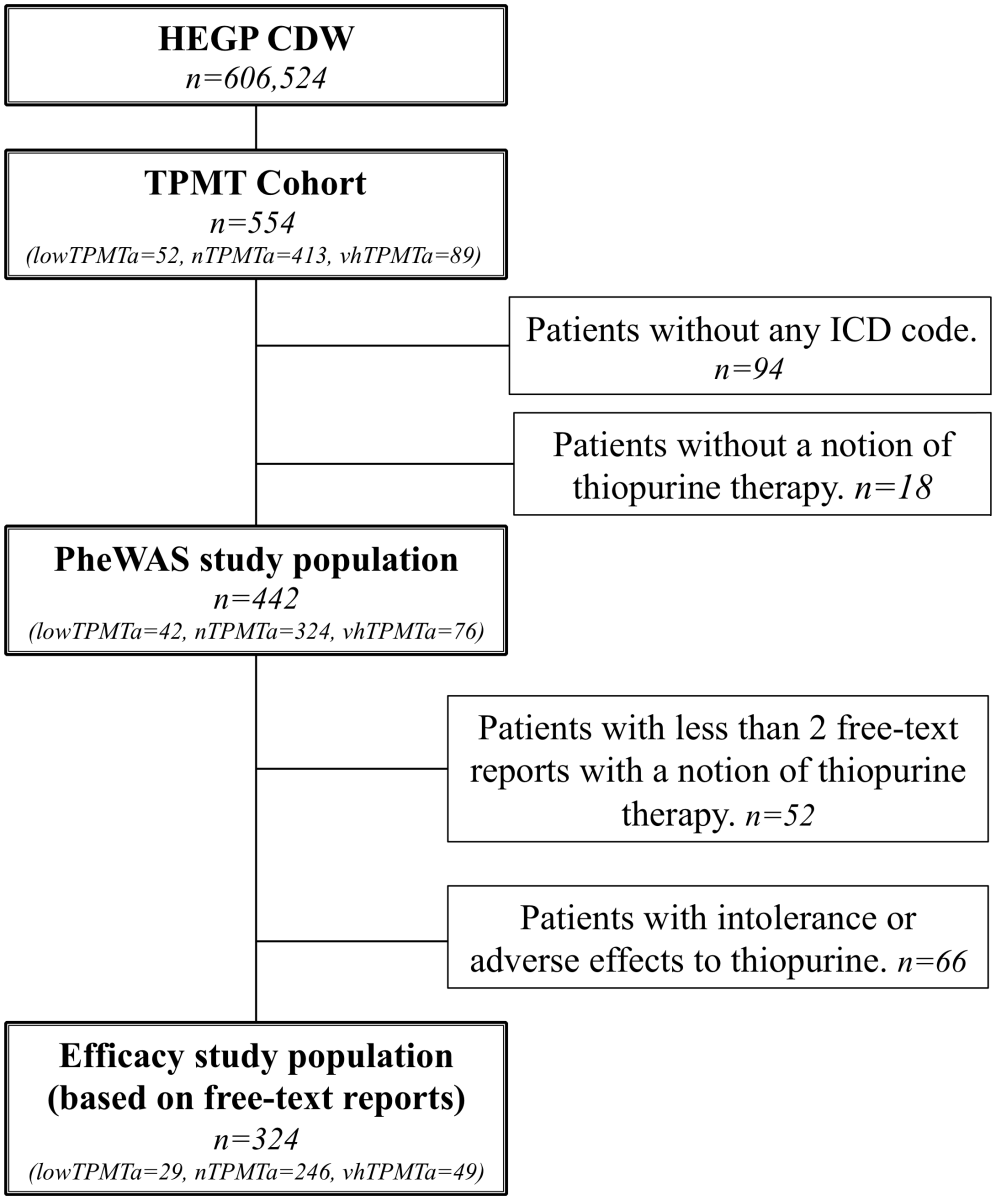
Flow chart. HEGP CDW: Clinical data warehouse from Hôpital Européen Georges Pompidou, France. TPMT Cohort: patients with a thiopurine S-methyltransferase (TPMT) activity assessment in HEGP between January 2000 and July 2013. ICD: International Statistical Classification of Diseases and Related Health Problems. PheWAS: phenome-wide association study.

A total of 554 patients (*TPMT cohort*) underwent a TPMTa assessment. Of these patients, 460 had ICD-10 codes and at least one biological test result, and a total of 442 patients, *i.e. study population*, had also a notion of thiopurine treatment in their EHR. (Figure 3, Figure S6) These 442 EHRs included 10,252 ICD-10 occurrences and 72,711 results of the selected biological tests (Table 1). Of these patients, 324, representing 6,183 free-text reports, were included in the thiopurine efficacy validation analysis, after exclusion of the patients having less than two reports with a notion of thiopurine therapy and patients with an adverse effect or intolerance to thiopurines. (Figure 3) Known indications for thiopurine therapy, *e.g.*, Crohn's disease (OR, 699.6; 95%CI, 343.7–1,600, p = 1.73E-263) or ulcerative colitis (OR, 583.1; 95%CI, 237.9–1,843, p = 1.5E-144) and their consequences were significantly associated with the *TPMT cohort* versus hospital population (Table S3). No patient with leukemia or an associated pathology were found in the analysis, as there is no hematologic department at HEGP.

### PheWAS analysis

#### ICD groupings

Using our ICD-10 based aggregation, the 1,016 distinct ICD-10 codes occurring in the *study population* EHRs resulted in 156 distinct aggregated codes, including 83 codes with at least 5 occurrences. (Table S4) ICD-9-CM mapping aggregation led to 289 distinct aggregated codes, including 94 codes with at least 5 occurrences. (Table S5) These 156 and 289 aggregated codes represent respectively 59% and 37% of the aggregated classifications.

**Figure 4:**
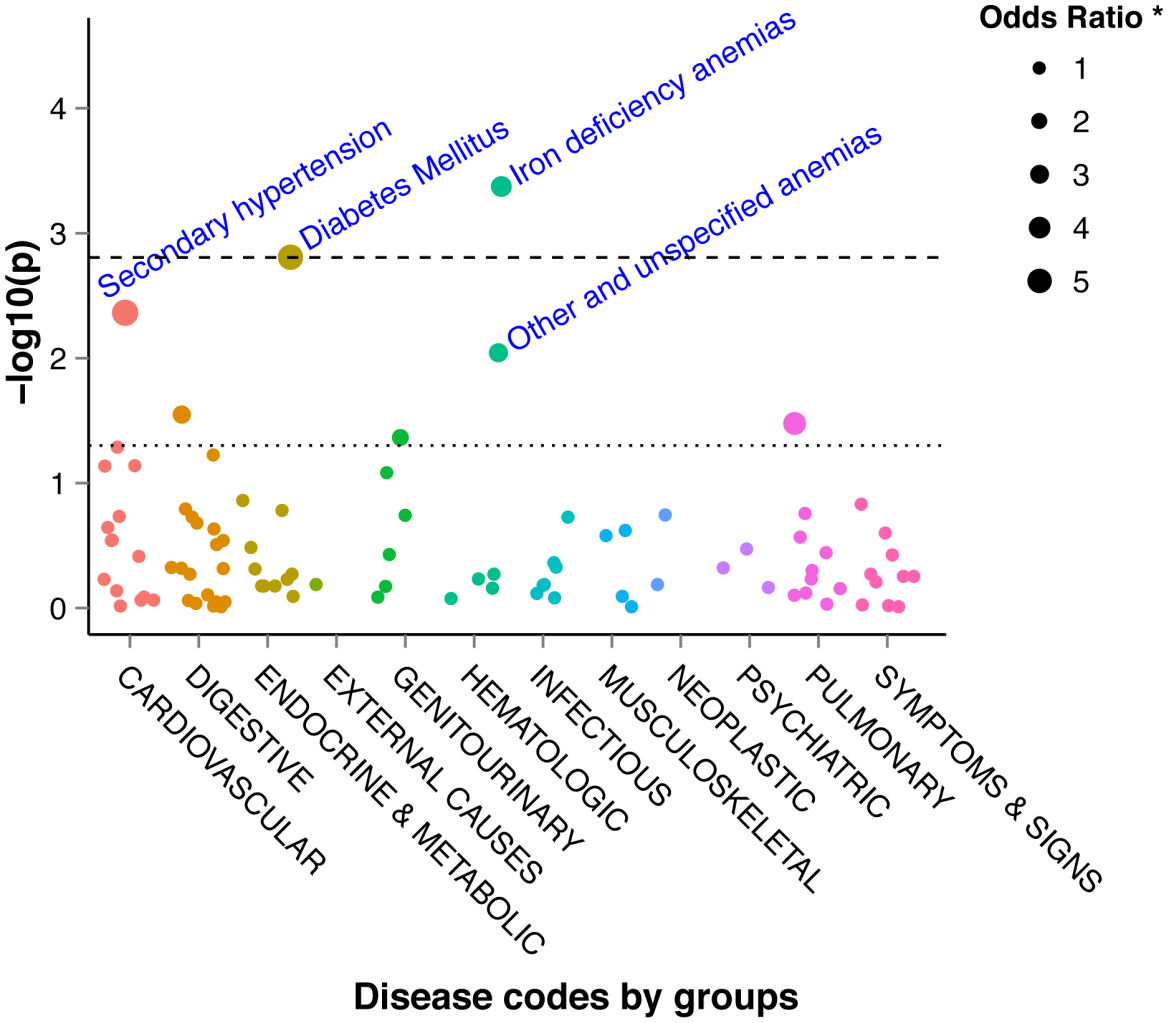
Manhattan plot of −log10 (P-values) for the 771 ICD-9-CM based aggregated codes between very high TPMT activity patients and other TPMT activity patients. ICD-9-CM: International classification of diseases 9 clinically modified; TPMT: thiopurine *S*-methyltransferase. The dotted line represents a P-value of 0.05 and the dashed line represents the FDR corrected level of significance for q = 0.2.

**Figure 5:**
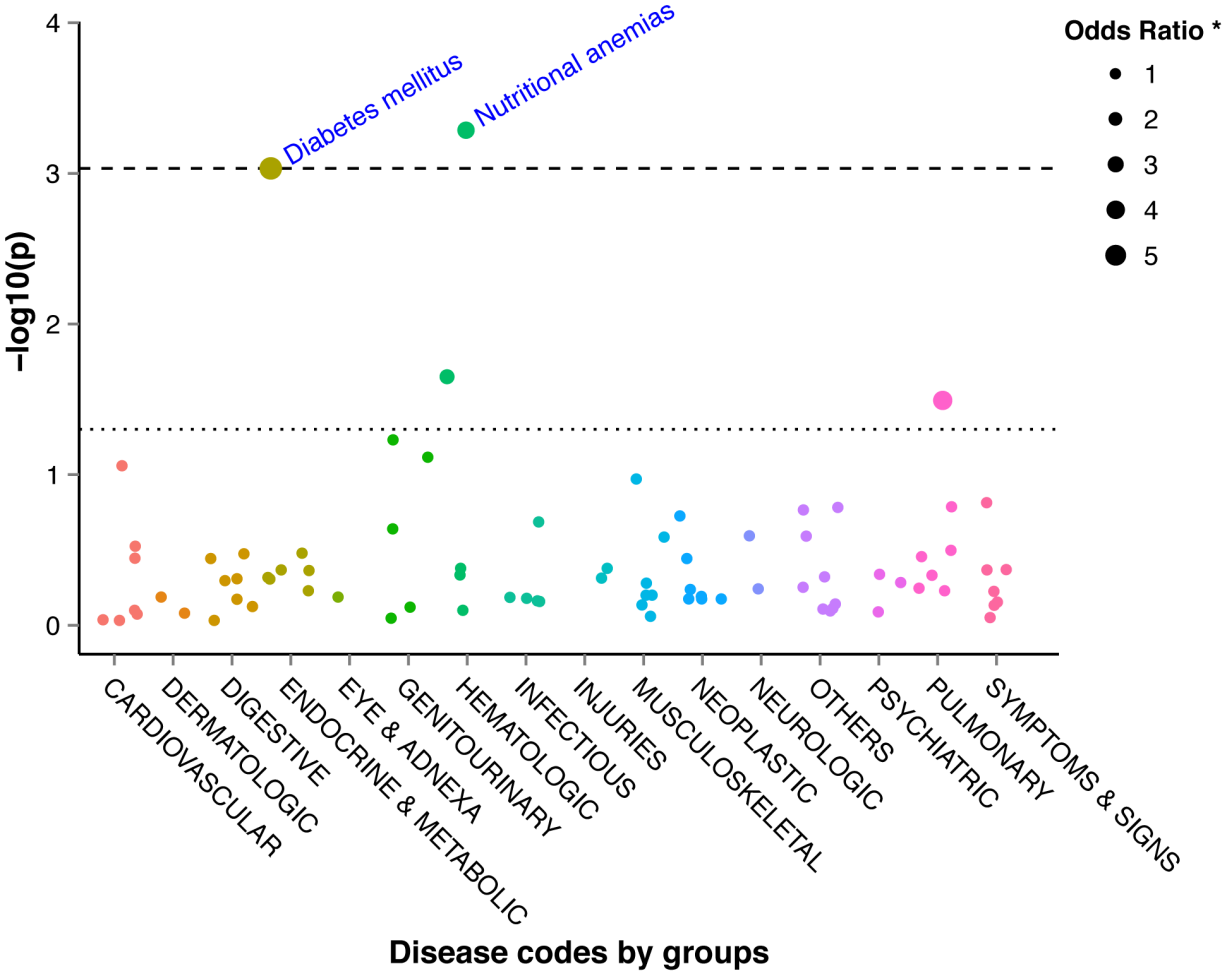
Manhattan plot of −log10 (P-values) for the 256 ICD-10 based aggregated codes between very high TPMT activity patients and other TPMT activity patients. ICD-10: International classification of diseases 10; TPMT: thiopurine *S*-methyltransferase. The dotted line represents a P-value of 0.05 and the dashed line represents the FDR corrected level of significance for q = 0.2.

In the vhTPMTa versus other TPMTa analysis, two significant codes for ICD-10 based aggregation were found: diabetes mellitus (p = 0.0009) and nutritional anemia (p = 0.0005). These results agreed with the ICD-9-CM mapping codes (p = 0.0004 and p = 0.0015, respectively). (Figures 4, 5, Tables S6, S7) These results remained significant after FDR multitesting evaluation for the two aggregation methods. (Tables S6, S7) The distribution of p-values did not show any systemic bias according to q-q plots. (Figure S3)

In the lowTPMTa versus other TPMTa analysis, no grouping showed statistically significant results after FDR correction. (Tables S8, S9, Figures S4, S5)

#### Biological test results

**Figure 6:**
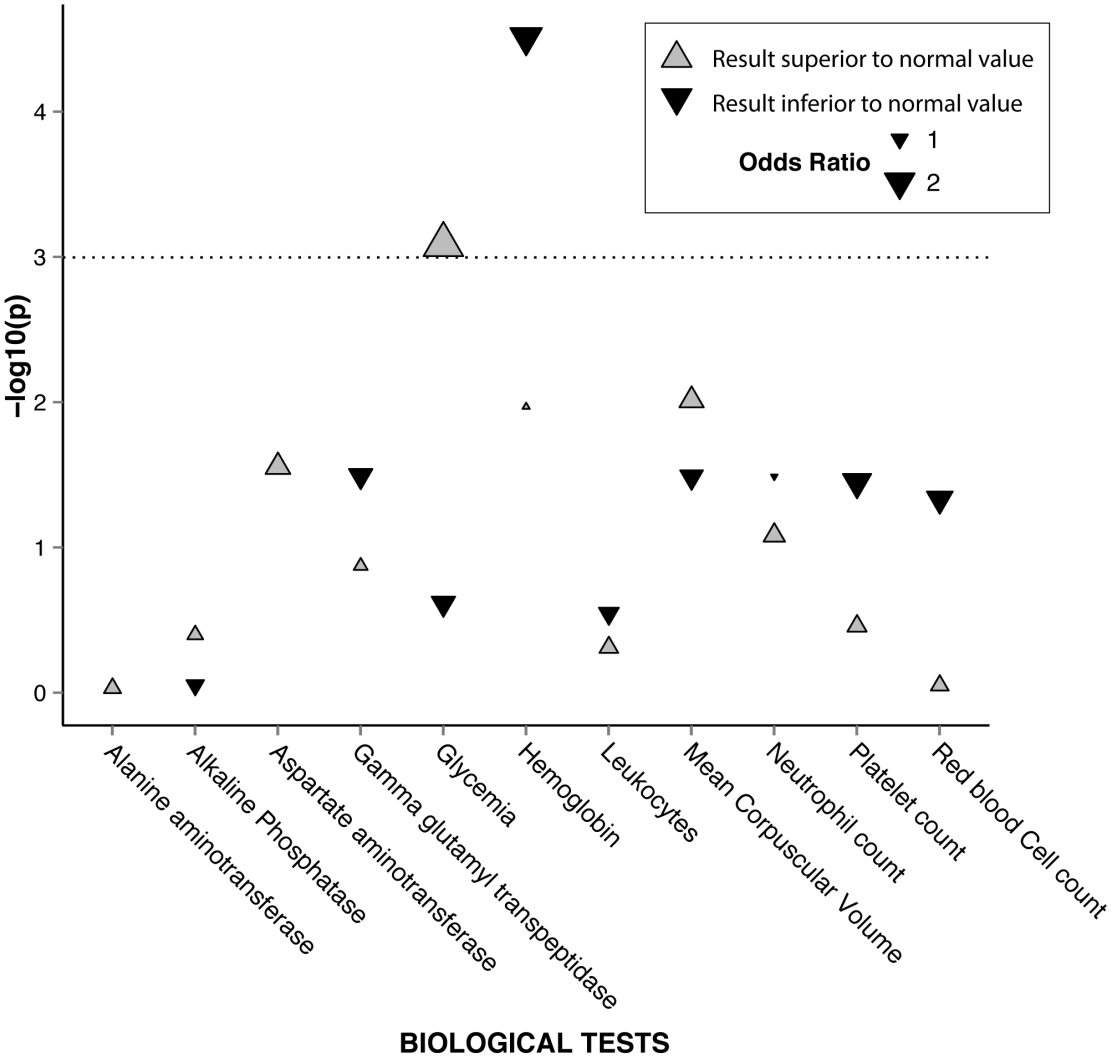
Pseudo-Manhattan plot of −log10 (P-values) for the 11 biological tests between very high TPMT activity patients and other TPMT activity patients. Using the global approach, a high-value case, resp. low-value case, is defined as at least one occurrence of a biological test result above, resp. below, the high or low threshold. Low-value case analyses have not been performed on alanine aminotransferase, aspartate aminotransferase and gamma glutamyl-transpeptidase test results, as a low threshold is not relevant for these tests. The dotted line represents a P-value of 0.05. Grey triangles represent the results above the high threshold and black triangles represent the results below the low threshold.

With the “global approach”, the proportion of patients with at least one episode of moderate to severe biological anemia was higher in the vhTPMTa group than in the other TPMTa group: 40.8% versus 26.1% (OR, 1.9; 95%CI, 1.2–3.3;p = 0.01). (Table 2, Figure 6) Analyzing the same groupings, we also found that 13.6% of vhTPMTa patients had an episode of hyperglycemia versus 5.9% in the other TPMTa group (OR, 2.48; 95%CI, 1–6.1;p = 0.046) (Table 3). The “frequency-based approach” confirmed that the mean frequency of moderate to severe biological anemia episodes was higher in the vhTPMTa group than in the other TPMTa group: 18% versus 9% of encounters (p = 0.01). (Table 2) On the other hand, there was no statistically significant difference in the frequency of encounters with hyperglycemia between the two groups. (Table 3) With respect to neutropenia, it was interesting to note that there was no difference between the two groups using the global approach. However, the “frequency-based approach” identified a lower rate of neutropenia in the vhTPMTa group than in the other TPMTa groups (Table 2).

**Table 2:** Results of the low-value case biological test analyses between very high TPMT activity patients and other patients with normal and low TPMT activity.

Biological tests	Global approach				Frequency-based approach		
	vhTPMTa n = 76 (%)	nTPMTa + lowTPTMa n = 366 (%)	Odds Ratio [95%CI]	Unadjusted p-value	vhTPMTa encounter frequency	nTPMTa+lowTPTMa encounter frequency	Unadjusted p-value
Leukocyte count	20/76 (26.3)	84/360 (23.3)	1.2 [0.7–2.1]	0.58	0.13	0.09	0.22
**Neutrophil count**	1/76 (1.3)	16/357 (4.5)	0.3 [0–2.2]	0.22	**0.003**	**0.014**	**0.041**
Red blood cell count	68/76 (89.5)	304/360 (84.4)	1.6 [0.7–3.4]	0.26	0.78	0.71	0.13
**Hemoglobin**	**31/76 (40.8)**	**94/360 (26.1)**	**1.9 [1.2–3.3]**	**0.01**	**0.18**	**0.09**	**0.01**
Mean corpuscular volume	40/76 (52.6)	162/360 (45)	1.4 [0.8–2.2]	0.22	0.4	0.31	0.12
Platelet count	7/76 (9.2)	20/360 (5.6)	1.7 [0.7–4.2]	0.23	0.03	0.02	0.67
Glycemia	59/63 (93.7)	273/299 (91.3)	1.4 [0.5–4.2]	0.54	0.78	0.76	0.65
Alkaline phosphatase	25/71 (35.2)	117/336 (34.8)	1 [0.6–1.7]	0.95	0.19	0.18	0.81

Global approach: a low-value case is defined as at least one occurrence, over the study period, of biological test result below the low threshold defined in Table 1. Frequency-based approach: for a given patient, the frequency of low-value encounters is defined as the number of encounters with at least one occurrence below the low threshold divided by the number of encounters (mean low-value encounter frequencies are reported).

Low-value case analyses have not been performed on alanine aminotransferase, aspartate aminotransferase and gamma glutamyl-transpeptidase test results, as a low threshold is not relevant for these tests.

TPMTa: thiopurine *S*-methyltransferase activity. lowTPMTa: low TPMTa (<8.5 nmol/h/mL red blood cells); vhTPMTa: very high TPMTa (≥15.0 nmol/h/mL red blood cells); nTPMTa: normal TPMTa (in between).

**Table 3:** Results of the high-value case biological test analyses between very high TPMT activity patients and other patients with normal and low TPMT activity.

Biological tests	Global approach				Frequency-based approach		
	vhTPMTa n = 76 (%)	nTPMTa+lowTPTMa n = 366 (%)	Odds Ratio [95%CI]	Unadjusted p-value	vhTPMTa encounter frequency	nTPMTa+lowTPTMa encounter frequency	Unadjusted p-value
Leukocyte count	52/76 (68.4)	239/360 (66.4)	1.1 [0.6–1.9]	0.733	0.41	0.37	0.41
Neutrophil count	41/76 (53.9)	171/357 (47.9)	1.27 [0.8–2.1]	0.339	0.24	0.2	0.3
Red blood cell count	5/76 (6.6)	23/360 (6.4)	1.03 [0.4–2.8]	0.951	0.01	0.02	0.22
**Hemoglobin**	2/76 (2.6)	27/360 (7.5)	0.33 [0.1–1.4]	0.14	**0.003**	**0.02**	**0.0008**
Mean corpuscular volume	34/76 (44.7)	128/360 (35.6)	1.47 [0.9–2.4]	0.134	0.29	0.21	0.08
Platelet count	35/76 (46.1)	155/360 (43.1)	1.13 [0.7–1.9]	0.632	0.25	0.2	0.32
**Glycemia**	**8/59 (13.6)**	**17/286 (5.9)**	**2.48 [1–6.1]**	**0.046**	0.06	0.03	0.24
Alkaline phosphatase	31/71 (43.7)	156/336 (46.4)	0.89 [0.5–1.5]	0.671	0.31	0.28	0.53
Alanine aminotransferase	19/72 (26.4)	91/342 (26.6)	0.99 [0.6–1.8]	0.969	0.09	0.11	0.67
Aspartate aminotransferase	16/72 (22.2)	55/342 (16.1)	1.49 [0.8–2.8]	0.211	0.06	0.06	0.81
Gamma glutamyl-transpeptidase	15/72 (20.8)	85/335 (25.4)	0.77 [0.4–1.4]	0.418	0.11	0.13	0.58

Global approach: a low-value case is defined as at least one occurrence, over the study period, of biological test result below the low threshold defined in Table 1. Frequency-based approach: for a given patient, the frequency of low-value encounters is defined as the number of encounters with at least one occurrence below the low threshold divided by the number of encounters (mean low-value encounter frequencies are reported).

TPMTa: thiopurine *S*-methyltransferase activity. lowTPMTa: low TPMTa (<8.5 nmol/h/mL red blood cells); vhTPMTa: very high TPMTa (≥15.0 nmol/h/mL red blood cells); nTPMTa: normal TPMTa (in between).

There were no differences between groups when comparing lowTPMTa versus other TPMTa group using the “global approach”. However, the “frequency-based approach” showed a lower frequency of leucopenia (3.7% versus 10%, p = 0.02) and neutropenia (0.9% versus 2.7%, p = 0.01) in the lowTPMTa group compared to other TPMTa group.(Tables S10, S11)

### Event-free survival analysis

**Figure 7:**
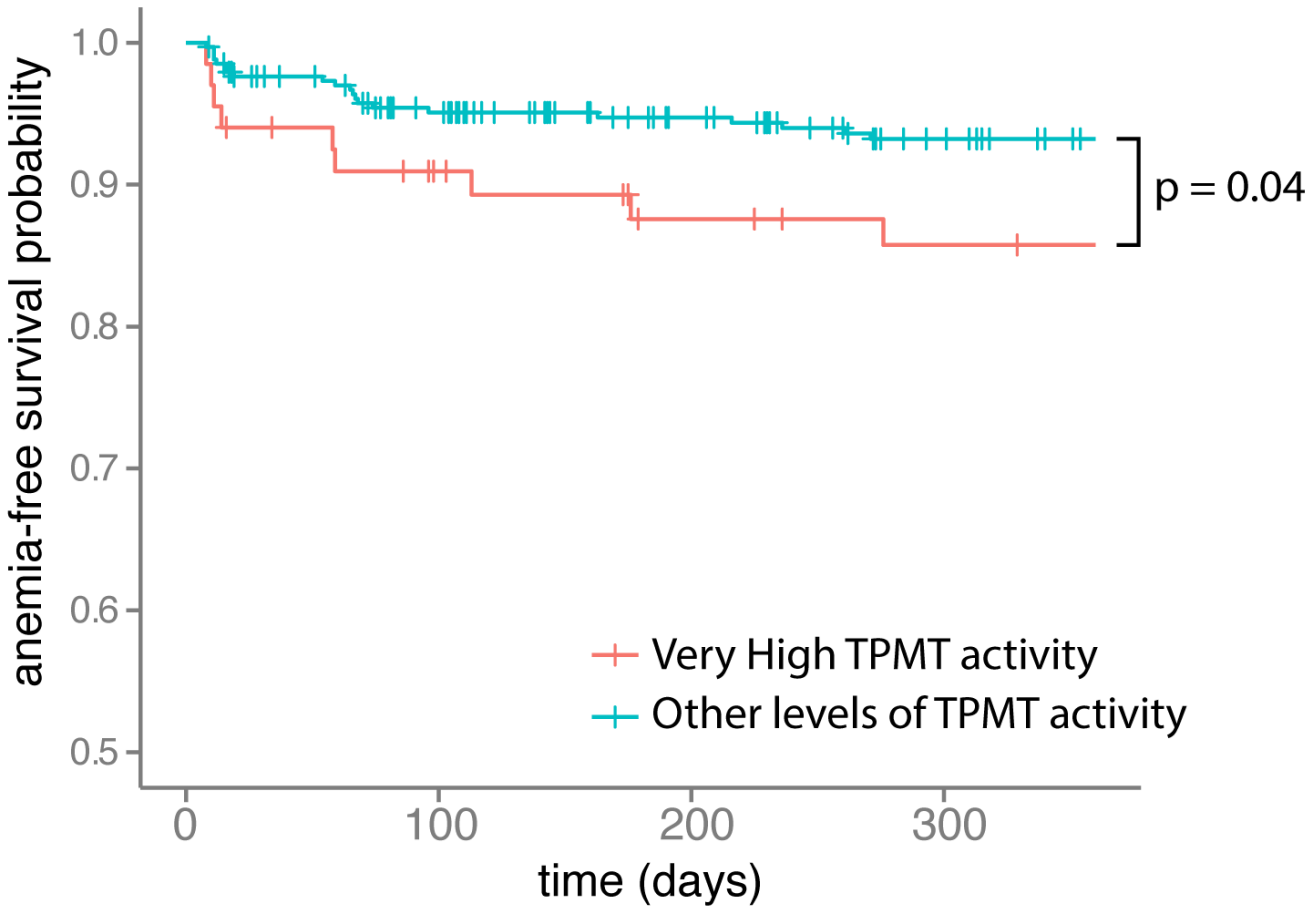
Kaplan-Meier survival analysis of the time without anemia after starting thiopurine therapy in very high TPMT activity patients versus other TPMT activity patients. TPMT: thiopurine *S*-methyltransferase. Analysis based on biological test results. Anemia was censored for hemoglobin test results below 9 g/100 mL. All events occurring within the first week after starting thiopurine therapy were excluded from the analysis. Follow-up was censored after 360 days. A log-rank test was used for this analysis.

Event-free survival was evaluated for anemia and hyperglycemia. It showed that patients with vhTPMTa had a significant risk to have earlier anemia episodes than others (p = 0.04). (Figure 7) Regarding the development of hyperglycemia, there was no difference between the groups.

### Thiopurine efficacy analysis

The efficacy analysis, based on free-text reports, showed 30.6% (15/49) of thiopurine therapy failure in the vhTPMTa group versus 13.1% (36/275) in the other TPMTa group (OR, 2.91; 95%CI, 1.33–6.17; p = 0.0045). After adjustment for sex and age in a logistic regression model, we found an adjusted OR of 3.11 (95%CI, 1.61–6.04; p = 0.0007).

## Discussion

This study demonstrates the feasibility and benefits of performing a PheWAS on a quantitative trait. Two independent approaches, based on (i) ICD codes and (ii) biological test results, were used to discover pathophysiological features potentially associated with this quantitative trait. In this manner, findings can be cross-validated: the phenotypes extracted from diagnosis codes were confirmed by the biological test results. By this way and using a quantitative trait in the context of pharmacogenomics we discovered new potential associations between TPMTa related to thiopurine treatment and clinical data.

To our knowledge, this is the first PheWAS performed using data encoded with ICD-10 classification, as previously published PheWAS were based on ICD-9-CM. The consistency in the results found between the two aggregation methods –the ICD-10-based method and the mapping between ICD-9-CM and ICD-10– demonstrates the feasibility of PheWAS using ICD-10. In our study population and using the ICD code distribution described above, ICD-9-CM based PheWAS codes resulted in more informative phenotypes than the ICD-10 based. Thus, it appears that ICD code aggregation level, *i.e.* the number of code groups, needs to be optimized according to the size of the population. For example, in a larger population, it may be more appropriate to use a fine grained aggregation based on the 3 digit codes of ICD-10, resulting in more accurate phenotypes.

The use of a CDW gives the opportunity to combine data from six heterogeneous sources: demographic data from administrative records, diagnosis codes from the billing system, biological test results, drug prescriptions from the CPOE system, free text reports, and clinical data from structured questionnaires. The clinical interpretation of patient condition by the physician, encoded with ICD codes, and the biological test results, extracted from the laboratory result server, were confronted. Drug prescriptions were extracted directly from the structured data issued by CPOE, structured questionnaires and from free-text reports. The close relationship between thiopurine drug prescriptions and TPMTa assays for therapeutic management was taken into account by incorporating temporal data for this study. Therefore, we restricted our analyses to the events following the initiation of thiopurine therapy.

In addition to patient selection based on TPMTa, biological test results were employed to validate the phenotypes obtained from ICD codes analysis. Thus, we assessed the feasibility of expanding PheWAS to another type of data from the CDW. In that aim, classification algorithms were developed to transform continuous test results into discrete classes using value and frequency thresholds. Such algorithms could benefit from semantic web technologies [45], because description logic includes reasoning capabilities. First, the patient's history was considered globally to compare the proportion of patients with an occurrence of abnormal biological test result between groups. In a second step, we analyzed the number of episodes for a specific biological abnormality, allowing us to compare event frequencies between TPMTa groups.

From a clinical point of view, the analyses using ICD-9-CM- or ICD-10-based groupings and biological test results are consistent, resulting in more frequent anemia in vhTPMTa patients than in other patients. In IBD, anemia is frequently observed and has a multifactorial etiology such as chronic inflammation or iron-deficiency caused by enteric bleeding [46]. In addition, myelosuppressive drugs such as thiopurines can cause anemia [47]. In our study, the strong association between iron-deficiency anemia – observed by ICD codes and hemoglobin test results – and vhTMPTa could reflect more active disease in these patients. Moreover, evaluation of the anemia-free duration showed earlier episodes of anemia in the vhTPMTa group compared to other patients. Finally, thiopurine efficacy analysis on free-text reports showed a three times more therapy failure occurrences in the vhTPMTa group, in relation with anemia episodes and an active disease. Besides, an over-representation of diabetes mellitus, identified by ICD-9-CM and ICD-10 mapping analyses, has been observed in patients with a vhTPMTa. This result has been confirmed by glycemia test result analyses with more patients having hyperglycemia. Onset of type 2 diabetes or glucose intolerance could result from a sustained steroid therapy secondary to thiopurine resistance and active disease in vhTPMTa patients. This finding is strengthened by the weak association with secondary hypertension also known as a steroid adverse effect. Finally, the higher risk of thiopurine therapy failure in vhTPMTa patients, highlighted by free-text report analysis, is in agreement with sustained steroid therapy, according to IBD therapeutic management. Altogether, these findings suggested that patients with vhTPMTa could have more active disease than the others, leading to more frequent anemia episodes despite thiopurine therapy. These patients may benefit from more intensive thiopurine therapy to maintain remission, spare steroids and lessen common adverse effects.

As a limit of our PheWAS study, the study design does not distinguish the effect of vhTPMTa itself from a drug effect. A possible approach to assess this point would be to perform a PheWAS on patients with a TPMTa assessment but without thiopurine therapy. However, according to TPMTa testing indication, *i.e.* before starting a thiopurine therapy to screen TPMT-deficient patients, the HEGP CDW did not contain data to process such an analysis. Systematic TPMTa determination for inpatients, in a context a large DNA biobanking could be valuable for analyzing the impact of vhTPMTa on clinical phenotypes.

The number of patients in our study (n = 442) is relatively small. Previously published PheWAS were mainly based on pooled data or large population based cohorts [7], [13], [14]. However, despite the size of our study, we obtained statistically significant results and supported by a clinical/biological cross-validation. This cross-validation was followed by a manual in-depth analysis of free-text reports to evaluate the validity of our initial conclusions. Regarding multiple testing issues, Denny *et al.* used a Bonferonni correction but estimated that it might be too restrictive [7], [42], [48]. We decided to use FDR because of its tolerance towards auto-correlated tests [49]. Given the cross-validation process based on the biological test results: (i) we did not exclude PheWAS codes with small numbers of cases from our analysis as in previous studies; (ii) and we considered the patients who had at least one occurrence of the ICD code p as having the phenotype p, whereas previous studies considered patients as cases when the ICD code was present more than once in the patient record (a minimum of two or even four occurrences of the same code) [8], [11], [12], [16].

To be used as a selection criterion, a quantitative trait should be stable over the period of phenotype analysis. As all enzymes, TPMT can be influenced by physiological factors (*e.g.*, pregnancy) or co-treatments [29], [50]. In our study, TPMTa was stable over the analysis period. To extend this method to other quantitative traits, this stability over time must be checked.

Regarding our ICD and biological test result analyses, it could be valuable to extend it to other retrospective cohorts or CDW. Finally, the implementation of a large prospective study, including patients treated by thiopurine according to their TPMTa, could help to confirm our findings regarding vhTPMTa and thiopurine therapy failure associated with steroid side effects, and to develop further research.

We described here an original method to perform a PheWAS analysis on a quantitative trait, TPMTa, using both ICD-10 diagnosis codes and biological test results to identify associated phenotypes. This study highlighted a potential association between very high TPMT activity and signs that could be associated with a failure of thiopurine therapy and sustained steroid requirements in IBD patients. In the field of pharmacogenomics, PheWAS may allow the description of new subgroups of patients who need personalized clinical and therapeutic management.

## Supporting Information

Figure S1 **Comparison between Genome Wide Association Studies (GWAS) and Phenome Wide Association Studies (PheWAS).** SNP: single nucleotide polymorphism. A. GWAS: a group of patients with a selected phenotype (*i.e.* disease) is compared to a control group. All the genomic data available are screened to find systematic genomic differences between the groups. B. PheWAS: a group of patients with a selected allele or SNP on a particular gene is compared to a control group with different alleles on the same gene. All the phenotypic data available are screened to find systematic phenotypic differences between the groups.
(EPS)

Figure S2 **Schematic representation of the discretization of quantitative biological test results for one single patient.** A. Global approach: a patient is considered as a high-value case if he has at least one occurrence of a biological test result above the high threshold. B. Frequency-based approach: the frequency of high-value encounters is defined as the number of encounters with at least one occurrence above the high threshold divided by the number of encounters.
(EPS)

Figure S3 **q-q plots of p-values from phenome-wide association study.** Left: q-q plot of p-values from the analysis of ICD codes with the ICD-10 based aggregation. Right: q-q plot of p-values from the analysis of ICD codes with the ICD-9-CM mapping based aggregation. The red line represents the normal distribution.
(EPS)

Figure S4 **Manhattan plot of Phenome-wide association study (PheWAS) between low TPMT activity patients and other TPMT activity patients for the ICD-9-CM mapping aggregation.** Groups of ICD codes are represented by dots. Results of association tests (logistic regressions) are represented vertically (−log10(p-value)). The dotted line indicates p = 0.05. The dashed line indicates the FDR corrected level of significance for q = 0.2. When the p-value is under 0.05, the dot size represents the level of the odds-ratio. TPMTa: thiopurine *S*-methyltransferase activity. Low TPMTa: <8.5 nmol/h/mL red blood cells; Very high TPMTa: ≥15.0 nmol/h/mL red blood cells; Normal TPMTa: in between.
(EPS)

Figure S5 **Manhattan plot of Phenome-wide association study (PheWAS) between low TPMT activity patients and other TPMT activity patients for the ICD-10 based aggregation.** Groups of ICD codes are represented by dots. Results of association tests (logistic regressions) are represented vertically (−log10(p-value)). The dotted line indicates p = 0.05. The dashed line indicates the FDR corrected level of significance for q = 0.2. When the p-value is under 0.05, the dot size represents the level of the odds-ratio. TPMTa: thiopurine *S*-methyltransferase activity. Low TPMTa: <8.5 nmol/h/mL red blood cells; Very high TPMTa: ≥15.0 nmol/h/mL red blood cells; Normal TPMTa: in between.
(EPS)

Figure S6 **Distribution of TPMT activity in the study population (n = 442).** TPMT: thiopurine *S*-methyltransferase; RBC: red blood cells.
(EPS)

Table S1 **Thresholds for biological test result analyses.** Thresholds have been defined according to the normal value ranges of the hospital laboratory. One test result occurrence below the low or above the high threshold defines a low-value case or a high-value case, respectively. * one neutrophil count below the low threshold of 1.0 G/L defines a neutropenia [51]. **one hemoglobin test result below the low threshold of 9.0 g/100 mL defines a moderate to severe biological anemia [52]. *** specially for glycemia, a high-value case (hyperglycemia) is defined by two test result occurrences above the high threshold [53].
(DOCX)

Table S2 **Thiopurine S-methyltransferase activity (TPMTa) for patients with multiple assays.** RBC: red blood cells. TPMTa: TPMT activity. Over the 51 patients that underwent more than one TPMTa assay, only one patient had results that could induce a change in groups. He was assigned to the normal TPMTa group (group from his first TPMTa assessment). For all the other patients, we considered that the TPMTa was stable over time.
(DOCX)

Table S3 **Results** of the preliminary phenome-wide association study on patients from TPMT cohort versus randomly selected patients from hospital clinical data warehouse. The ICD codes aggregation used was based on the 3-digit ICD-10 codes (2040 groups). Only the statistically significant results are reported here.
(DOCX)

Table S4 **Distribution of PheWAS Codes from ICD-10 based aggregation.**
(DOCX)

Table S5 **Distribution of PheWAS Codes from ICD-9-CM mapping aggregation.**
(DOCX)

Table S6 **Results** of the Phenome-wide association study (PheWAS) between very high TPMT activity patients and other TPMT activity patients for the ICD-9-CM mapping aggregation. The ICD-9-CM mapping aggregation corresponds to 771 groups of codes. Associations are assessed using logistic regression. Only PheWAS codes with a p-value<0.05 are reported here. The q value for false discovery rate (FDR) was q = 0.2. The p-value must be under the calculated FDR threshold to be considered as significant. TPMTa: thiopurine *S*-methyltransferase activity. Low TPMTa: <8.5 nmol/h/mL red blood cells; Very high TPMTa: ≥15.0 nmol/h/mL red blood cells; Normal TPMTa: in between.
(DOCX)

Table S7 **Results** of the Phenome-wide association study (PheWAS) between very high TPMT activity patients and other TPMT activity patients for the ICD-10 based aggregation. The ICD-10 based aggregation corresponds to 256 groups of codes. Only PheWAS codes with a p-value<0.05 are reported here. Associations are assessed using logistic regression. The q value for false discovery rate (FDR) was q = 0.2. The p-value must be under the calculated FDR threshold to be considered as significant. TPMTa: thiopurine *S*-methyltransferase activity. Low TPMTa: <8.5 nmol/h/mL red blood cells; Very high TPMTa: ≥15.0 nmol/h/mL red blood cells; Normal TPMTa: in between.
(DOCX)

Table S8 **Results** of the Phenome-wide association study (PheWAS) between low TPMT activity patients and other TPMT activity patients for the ICD-9-CM mapping aggregation. The ICD-9-CM mapping aggregation corresponds to 771 groups of codes. Only PheWAS codes with a p-value<0.05 are reported here. Associations are assessed using logistic regression. The q value for false discovery rate (FDR) was q = 0.2. The p-value must be under the calculated FDR threshold to be considered as significant. TPMTa: thiopurine *S*-methyltransferase activity. Low TPMTa: <8.5 nmol/h/mL red blood cells; Very high TPMTa: ≥15.0 nmol/h/mL red blood cells; Normal TPMTa: in between.
(DOCX)

Table S9 **Results** of the Phenome-wide association study (PheWAS) between low TPMT activity patients and other TPMT activity patients for the ICD10 based aggregation. The ICD-10 based aggregation corresponds to 256 groups of codes. Only PheWAS codes with a p-value<0.05 are reported here. Associations are assessed using logistic regression. The q value for false discovery rate (FDR) was q = 0.2. The p-value must be under the calculated FDR threshold to be considered as significant.
(DOCX)

Table S10 **Results** of the low-value case biological test analyses between low TPMT activity patients and other patients with normal and very high TPMT activity. Global approach: a low-value case is defined as at least one occurrence, over the study period, of biological test result below the low threshold defined in Table 1. Frequency-based approach: for a given patient, the frequency of low-value encounters is defined as the number of encounters with at least one occurrence below the low threshold divided by the number of encounters (mean low-value encounter frequencies are reported). Low-value case analyses have not been performed on alanine aminotransferase, aspartate aminotransferase and gamma glutamyl-transpeptidase test results, as a low threshold is not relevant for these tests.
(DOCX)

Table S11 **Results** of the high-value-case biological test analyses between low TPMT activity (lowTPMTa) patients and other patients. Global approach: a high-value case is defined as at least one occurrence, over the study period, of biological test result above the high threshold defined in Table 1. Frequency-based approach: for a given patient, the frequency of low-value encounters is defined as the number of encounters with at least one occurrence below the low threshold divided by the number of encounters (mean low-value encounter frequencies are reported). TPMTa: thiopurine S-methyltransferase activity. lowTPMTa: low TPMTa (<8.5 nmol/h/mL red blood cells); vhTPMTa: very high TPMTa (≥15.0 nmol/h/mL red blood cells); nTPMTa: normal TPMTa (in between).
(DOCX)
