# Supplementary material for: Phenome-Wide Association Studies on a Quantitative Trait: Application to TPMT Enzyme Activity and Thiopurine Therapy in Pharmacogenomics
Source: PLoS Comput Biol. 2013 Dec 26;9(12):e1003405. doi: 10.1371/journal.pcbi.1003405 (PMC3873228; doi:10.1371/journal.pcbi.1003405)
Supplement: Table S1 — Thresholds for biological test result analyses. Thresholds have been defined according to the normal value ranges of the hospital laboratory. One test result occurrence below the low or above the high threshold defines a low-value case or a high-value case, respectively. * one neutrophil count below the low threshold of 1.0 G/L defines a neutropenia [51]. **one hemoglobin test result below the low threshold of 9.0 g/100 mL defines a moderate to severe biological anemia [52]. *** specially for glycemia, a high-value case (hyperglycemia) is defined by two test result occurrences above the high threshold [53]. (DOCX) [file pcbi.1003405.s007.docx]

Table S1: Thresholds for biological test result analyses

| **Biological tests** | **Units** | **Thresholds** | |
| --- | --- | --- | --- |
|  |  | **Low** | **High** |
| **Leukocyte count** | **G/L** | 4.0 | 10.1 |
| **Neutrophil count *** | **G/L** | 1.0 | 10.0 |
| **Red blood cell count** | **T/L** | 4.4 | 5.5 |
| **Mean corpuscular volume** | **fL** | 84 | 96 |
| **Hemoglobin **** | **g/100 mL** | 9.0 | 16.1 |
| **Platelet count** | **G/L** | 100 | 450 |
| **Glycemia ***** | **mmol/L** | 3.3 | 11.1 |
| **Alkaline phosphatase** | **UI/L** | 42 | 80 |
| **Alanine aminotransferase** | **UI/L** | - | 70 |
| **Aspartate aminotransferase** | **UI/L** | - | 70 |
| **Gamma glutamyl-transpeptidase** | **UI/L** | - | 100 |

Thresholds have been defined according to the normal value ranges of the hospital laboratory. One test result occurrence below the low or above the high threshold defines a low-value case or a high-value case, respectively.

* one neutrophil count below the low threshold of 1.0 G/L defines a neutropenia. [52]

**one hemoglobin test result below the low threshold of 9.0 g/100 mL defines a moderate to severe biological anemia.[51]

*** specially for glycemia, a high-value case (hyperglycemia) is defined by two test result occurrences above the high threshold.[53]
