# Supplementary material for: Phenome-Wide Association Studies on a Quantitative Trait: Application to TPMT Enzyme Activity and Thiopurine Therapy in Pharmacogenomics
Source: PLoS Comput Biol. 2013 Dec 26;9(12):e1003405. doi: 10.1371/journal.pcbi.1003405 (PMC3873228; doi:10.1371/journal.pcbi.1003405)
Supplement: Table S2 — Thiopurine S-methyltransferase activity (TPMTa) for patients with multiple assays. RBC: red blood cells. TPMTa: TPMT activity. Over the 51 patients that underwent more than one TPMTa assay, only one patient had results that could induce a change in groups. He was assigned to the normal TPMTa group (group from his first TPMTa assessment). For all the other patients, we considered that the TPMTa was stable over time. (DOCX) [file pcbi.1003405.s008.docx]

| **Patient Number** | **First TPMTa assay**  **(nmol/h/mL RBC)** | **Second TPMTa assay**  **(nmol/h/mL RBC)** |
| --- | --- | --- |
| 1 | 7.9 | 8.4 |
| 2 | 9.8 | 11.9 |
| 3 | 9.0 | 11.1 |
| 4 | 10.8 | 10.0 |
| 5 | 13.3 | 14.7 |
| 6 | 15.4 | 15.5 |
| 7 | 17.3 | 19.6 |
| 8 | 10.1 | 13.7 |
| 9 | 9.7 | 14.8 |
| 10 | 13.5 | 10.2 |
| 11 | 11.5 | 10.8 |
| 12 | 14.3 | 9.3 |
| 13 | 13.6 | 14.2 |
| 14 | 12.3 | 9.8 |
| 15 | 11.2 | 12.1 |
| 16 | 12.3 | 10.4 |
| 17 | 13.8 | 14.5 |
| 18 | 15.3 | 17.6 |
| 19 | 8.8 | 11.8 |
| 20 | 11.8 | 11.4 |
| 21 | 12.5 | 13.0 |
| 22 | 11.8 | 12.4 |
| 23 | 9.7 | 8.4 |
| 24 | 11.0 | 13.6 |
| 25 | 13.9 | 11.5 |
| 26 | 13.2 | 10.1 |
| 27 | 6.8 | 7.2 |
| 28 | 9.5 | 10.9 |
| 29 | 9.7 | 9.7 |
| 30 | 7.4 | 6.0 |
| 31 | 7.5 | 6.3 |
| 32 | 12.7 | 12.8 |
| 33 | 12.8 | 12.9 |
| 34 | 12.3 | 10.0 |
| 35 | 9.4 | 14.3 |
| 36 | 11.3 | 9.3 |
| 36 | 11.1 | 9.1 |
| **37** | **10.2** | **15.9** |
| 38 | 12.3 | 11.7 |
| 39 | 11.4 | 11.7 |
| 40 | 13.1 | 11.7 |
| 41 | 13.2 | 11.9 |
| 42 | 14.7 | 14.3 |
| 43 | 14.1 | 14.0 |
| **Patient Number** | **First TPMTa assay**  **(nmol/h/mL RBC)** | **Second TPMTa assay**  **(nmol/h/mL RBC)** |
| 44 | 12.7 | 11.9 |
| 45 | 12.4 | 14.5 |
| 46 | 6.9 | 7.2 |
| 47 | 12.8 | 14.5 |
| 48 | 15.9 | 15.7 |
| 49 | 8.6 | 9.3 |
| 50 | 13.3 | 14.2 |
| 51 | 11.5 | 10.7 |
