# Supplementary material for: Phenome-Wide Association Studies on a Quantitative Trait: Application to TPMT Enzyme Activity and Thiopurine Therapy in Pharmacogenomics
Source: PLoS Comput Biol. 2013 Dec 26;9(12):e1003405. doi: 10.1371/journal.pcbi.1003405 (PMC3873228; doi:10.1371/journal.pcbi.1003405)
Supplement: Table S3 — Results of the preliminary phenome-wide association study on patients from TPMT cohort versus randomly selected patients from hospital clinical data warehouse. The ICD codes aggregation used was based on the 3-digit ICD-10 codes (2040 groups). Only the statistically significant results are reported here. (DOCX) [file pcbi.1003405.s009.docx]

| **Code** | **Name** | **Cases in TPMT cohort** | **Cases in hospital cohort** | **Odds-Ratio [95%CI]** | **p-value** |
| --- | --- | --- | --- | --- | --- |
| K50 | Crohn's disease [regional enteritis] | 290/366 (79.2) | 9/1652 (0.5) | 699.6  [343.7 - 1600] | 1.73E-263 |
| K51 | Ulcerative colitis | 138/214 (64.5) | 5/1648 (0.3) | 583.1  [237.9 - 1843] | 4.29E-145 |
| K52 | Other noninfective gastroenteritis and colitis | 45/121 (37.2) | 12/1655 (0.7) | 80.2  [39.8 - 174.2] | 1.87E-45 |
| K56 | Paralytic ileus and intestinal obstruction without hernia | 56/351 (16) | 15/1619 (0.9) | 20.2  [11.1 - 39.1] | 1.83E-30 |
| K61 | Abscess of anal and rectal regions | 34/329 (10.3) | 4/1608 (0.2) | 46.1  [16.3 - 180.3] | 5.84E-23 |
| K63 | Other diseases of intestine | 37/332 (11.1) | 8/1612 (0.5) | 25.1 [11.3 - 63] | 4.08E-22 |
| K60 | Fissure and fistula of anal and rectal regions | 26/321 (8.1) | 1/1605 (0.1) | 140.9  [23 - 5595.4] | 5.63E-20 |
| D50 | Iron deficiency anaemia | 46/443 (10.4) | 16/1668 (1) | 11.9  [6.6 - 22.8] | 9.02E-20 |
| R10 | Abdominal and pelvic pain | 55/430 (12.8) | 40/1647 (2.4) | 5.9 [3.8 - 9.2] | 4.00E-16 |
| M31 | Other necrotizing vasculopathies | 27/426 (6.3) | 4/1660 (0.2) | 28 [9.7 - 110.7] | 1.66E-15 |
| K62 | Other diseases of anus and rectum | 27/322 (8.4) | 11/1615 (0.7) | 13.3  [6.3 - 30.1] | 7.25E-14 |
| E43 | Unspecified severe protein-energy malnutrition | 25/412 (6.1) | 6/1650 (0.4) | 17.7 [7 - 53] | 3.96E-13 |
| O04 | Medical abortion | 2/444 (0.5) | 139/1666 (8.3) | 0 [0 - 0.2] | 1.09E-12 |
| Y43 | Adverse effects in the therapeutic use of primarily systemic agents | 18/432 (4.2) | 1/1659 (0.1) | 71.9  [11.3 - 2956] | 5.44E-12 |
| K92 | Other diseases of digestive system | 26/418 (6.2) | 14/1654 (0.8) | 7.8 [3.9 - 16.2] | 5.92E-10 |
| N17 | Acute renal failure | 36/430 (8.4) | 31/1637 (1.9) | 4.7 [2.8 - 8] | 1.51E-09 |
| K65 | Peritonitis | 22/443 (5) | 11/1667 (0.7) | 7.9 [3.6 - 18.1] | 1.47E-08 |
| E44 | Protein-energy malnutrition of moderate and mild degree | 24/411 (5.8) | 16/1660 (1) | 6.4 [3.2 - 13] | 2.04E-08 |
| J84 | Other interstitial pulmonary diseases | 16/441 (3.6) | 4/1658 (0.2) | 15.5 [5 - 64.2] | 2.39E-08 |
| A04 | Other bacterial intestinal infections | 13/428 (3) | 2/1661 (0.1) | 25.9  [5.8 - 236.4] | 6.74E-08 |
| D64 | Other anaemias | 14/422 (3.3) | 4/1648 (0.2) | 14.1  [4.4 - 59.1] | 2.43E-07 |
| A09 | Diarrhoea and gastroenteritis of presumed infectious origin | 16/431 (3.7) | 7/1666 (0.4) | 9.1 [3.5 - 26.4] | 4.64E-07 |
| E46 | Unspecified protein-energy malnutrition | 13/400 (3.3) | 4/1648 (0.2) | 13.8  [4.2 - 58.4] | 5.62E-07 |
| A41 | Other septicaemia | 22/441 (5) | 16/1660 (1) | 5.4 [2.7 - 11.1] | 5.76E-07 |
| K85 | Acute pancreatitis | 15/428 (3.5) | 6/1647 (0.4) | 9.9 [3.6 - 31.4] | 6.66E-07 |
| M45 | Ankylosing spondylitis | 9/441 (2) | 0/1667 (0) | - | 7.19E-07 |
| J99 | Respiratory disorders in diseases classified elsewhere | 8/421 (1.9) | 0/1604 (0) | - | 3.31E-06 |
| K75 | Other inflammatory liver diseases | 11/426 (2.6) | 3/1647 (0.2) | 14.5  [3.8 - 81.3] | 4.92E-06 |
| K90 | Intestinal malabsorption | 14/406 (3.4) | 8/1648 (0.5) | 7.3 [2.8 - 20.3] | 7.65E-06 |
| I80 | Phlebitis and thrombophlebitis | 18/433 (4.2) | 14/1644 (0.9) | 5 [2.3 - 11.1] | 1.02E-05 |
| N18 | Chronic renal failure | 30/424 (7.1) | 39/1645 (2.4) | 3.1 [1.9 - 5.2] | 1.12E-05 |
| K83 | Other diseases of biliary tract | 8/421 (1.9) | 1/1642 (0.1) | 31.7  [4.2 - 1400.7] | 2.11E-05 |
| K59 | Other functional intestinal disorders | 10/305 (3.3) | 6/1610 (0.4) | 9 [3 - 30.5] | 2.99E-05 |
| I77 | Other disorders of arteries and arterioles | 14/427 (3.3) | 9/1587 (0.6) | 5.9 [2.4 - 15.7] | 3.74E-05 |
| M30 | Polyarteritis nodosa and related conditions | 6/405 (1.5) | 0/1656 (0) | - | 5.59E-05 |
| N08 | Glomerular disorders in diseases classified elsewhere | 12/436 (2.8) | 7/1660 (0.4) | 6.7 [2.4 - 20.1] | 6.86E-05 |
| N01 | Rapidly progressive nephritic syndrome | 6/430 (1.4) | 0/1653 (0) | - | 7.53E-05 |
| I27 | Other pulmonary heart diseases | 9/435 (2.1) | 3/1648 (0.2) | 11.6 [  2.9 - 66.8] | 8.44E-05 |
| D69 | Purpura and other haemorrhagic conditions | 13/440 (3) | 9/1663 (0.5) | 5.6 [2.2 - 14.9] | 9.64E-05 |
| M80 | Osteoporosis with pathological fracture | 7/438 (1.6) | 1/1649 (0.1) | 26.7  [3.4 - 1199.2] | 1.13E-04 |
| R70 | Elevated erythrocyte sedimentation rate and abnormality of plasma viscosity | 7/436 (1.6) | 1/1632 (0.1) | 26.6  [3.4 - 1192.4] | 1.17E-04 |
| D12 | Benign neoplasm of colon, rectum, anus and anal canal | 20/436 (4.6) | 22/1615 (1.4) | 3.5 [1.8 - 6.8] | 1.49E-04 |
| R50 | Fever of unknown origin | 18/400 (4.5) | 20/1556 (1.3) | 3.6 [1.8 - 7.3] | 1.56E-04 |
| B96 | Other bacterial agents as the cause of diseases classified to other chapters | 13/437 (3) | 10/1662 (0.6) | 5.1 [2 - 13] | 1.71E-04 |
| S82 | Fracture of lower leg, including ankle | 0/444 (0) | 39/1657 (2.4) | 0 [0 - 0.4] | 1.89E-04 |
| M35 | Other systemic involvement of connective tissue | 8/407 (2) | 3/1659 (0.2) | 11 [2.6 - 65] | 2.01E-04 |
| R63 | Symptoms and signs concerning food and fluid intake | 10/392 (2.6) | 6/1542 (0.4) | 6.7 [2.2 - 22.5] | 2.60E-04 |
| K71 | Toxic liver disease | 8/423 (1.9) | 3/1647 (0.2) | 10.5 [2.5 - 62] | 2.64E-04 |
| D61 | Other aplastic anaemias | 7/415 (1.7) | 2/1646 (0.1) | 14.1  [2.7 - 139.7] | 3.17E-04 |
| R91 | Abnormal findings on diagnostic imaging of lung | 8/442 (1.8) | 3/1663 (0.2) | 10.2  [2.4 - 59.9] | 3.23E-04 |
| R11 | Nausea and vomiting | 10/385 (2.6) | 7/1614 (0.4) | 6.1 [2.1 - 19.1] | 3.35E-04 |
| D62 | Acute posthaemorrhagic anaemia | 13/421 (3.1) | 12/1656 (0.7) | 4.4 [1.8 - 10.5] | 3.88E-04 |
| K26 | Duodenal ulcer | 8/414 (1.9) | 4/1613 (0.2) | 7.9 [2.1 - 36.1] | 6.48E-04 |
| S61 | Open wound of wrist and hand | 0/441 (0) | 32/1580 (2) | 0 [0 - 0.4] | 7.23E-04 |
| E78 | Disorders of lipoprotein metabolism and other lipidaemias | 21/427 (4.9) | 161/1625 (9.9) | 0.5 [0.3 - 0.8] | 7.86E-04 |
| K91 | Postprocedural disorders of digestive system, not elsewhere classified | 8/400 (2) | 5/1645 (0.3) | 6.7 [1.9 - 26.1] | 1.02E-03 |
| D86 | Sarcoidosis | 7/439 (1.6) | 3/1666 (0.2) | 9 [2 - 54.1] | 1.09E-03 |
| K93 | Disorders of other digestive organs in diseases classified elsewhere | 5/397 (1.3) | 1/1641 (0.1) | 20.9  [2.3 - 984.5] | 1.38E-03 |
| M33 | Dermatopolymyositis | 4/403 (1) | 0/1656 (0) | - | 1.45E-03 |
| D63 | Anaemia in chronic diseases classified elsewhere | 10/418 (2.4) | 9/1653 (0.5) | 4.5 [1.6 - 12.5] | 1.61E-03 |
| T45 | Poisoning by primarily systemic and haematological agents, not elsewhere classified | 6/443 (1.4) | 2/1625 (0.1) | 11.1 [2 - 113.1] | 1.77E-03 |
| M05 | Seropositive rheumatoid arthritis | 5/430 (1.2) | 1/1655 (0.1) | 19.4  [2.2 - 916.2] | 1.82E-03 |
| D84 | Other immunodeficiencies | 5/437 (1.1) | 1/1664 (0.1) | 19.2  [2.1 - 906.3] | 1.90E-03 |
| L40 | Psoriasis | 4/444 (0.9) | 0/1668 (0) | - | 1.93E-03 |
| J96 | Respiratory failure, not elsewhere classified | 29/442 (6.6) | 52/1656 (3.1) | 2.2 [1.3 - 3.5] | 1.95E-03 |
| M60 | Myositis | 4/444 (0.9) | 0/1653 (0) | - | 1.99E-03 |
| T81 | Complications of procedures, not elsewhere classified | 15/429 (3.5) | 19/1628 (1.2) | 3.1 [1.4 - 6.4] | 2.08E-03 |
| Y42 | Adverse effects in the therapeutic use of hormones and their synthetic substitutes and antagonists, not elsewhere classified | 6/420 (1.4) | 3/1661 (0.2) | 8 [1.7 - 49.7] | 3.15E-03 |
| R74 | Abnormal serum enzyme levels | 6/435 (1.4) | 3/1634 (0.2) | 7.6 [1.6 - 47.1] | 3.93E-03 |
| K73 | Chronic hepatitis, not elsewhere classified | 5/420 (1.2) | 2/1646 (0.1) | 9.9  [1.6 - 104.2] | 4.96E-03 |
| B95 | Streptococcus and staphylococcus as the cause of diseases classified to other chapters | 8/432 (1.9) | 7/1659 (0.4) | 4.4 [1.4 - 14.5] | 5.07E-03 |
| S66 | Injury of muscle and tendon at wrist and hand level | 1/442 (0.2) | 33/1581 (2.1) | 0.1 [0 - 0.6] | 5.13E-03 |
| M06 | Other rheumatoid arthritis | 5/430 (1.2) | 2/1656 (0.1) | 9.7  [1.6 - 102.4] | 5.29E-03 |
| M25 | Other joint disorders, not elsewhere classified | 7/444 (1.6) | 5/1645 (0.3) | 5.2  [1.4 - 21.1] | 5.46E-03 |
| B37 | Candidiasis | 5/442 (1.1) | 2/1666 (0.1) | 9.5  [1.5 - 100.2] | 5.72E-03 |
| N41 | Inflammatory diseases of prostate | 5/443 (1.1) | 2/1654 (0.1) | 9.4 [1.5 - 99.2] | 5.92E-03 |
| E66 | Obesity | 17/444 (3.8) | 122/1665 (7.3) | 0.5 [0.3 - 0.9] | 6.97E-03 |
| N00 | Acute nephritic syndrome | 4/428 (0.9) | 1/1654 (0.1) | 15.6 [1.5 - 765] | 7.39E-03 |
| G62 | Other polyneuropathies | 4/441 (0.9) | 1/1666 (0.1) | 15.2  [1.5 - 747.8] | 7.91E-03 |
| B44 | Aspergillosis | 4/441 (0.9) | 1/1665 (0.1) | 15.2  [1.5 - 747.3] | 7.93E-03 |
| N02 | Recurrent and persistent haematuria | 3/427 (0.7) | 0/1653 (0) | - | 8.60E-03 |
| M13 | Other arthritis | 3/428 (0.7) | 0/1654 (0) | - | 8.64E-03 |
| F43 | Reaction to severe stress, and adjustment disorders | 3/434 (0.7) | 0/1652 (0) | - | 8.96E-03 |
| B25 | Cytomegaloviral disease | 3/438 (0.7) | 0/1665 (0) | - | 8.99E-03 |
| D72 | Other disorders of white blood cells | 3/437 (0.7) | 0/1661 (0) | - | 8.99E-03 |
| M54 | Dorsalgia | 7/443 (1.6) | 6/1664 (0.4) | 4.4 [1.3 - 16.1] | 9.11E-03 |
| E86 | Volume depletion | 6/412 (1.5) | 4/1468 (0.3) | 5.4 [1.3 - 26.1] | 1.00E-02 |
| R57 | Shock, not elsewhere classified | 14/396 (3.5) | 22/1558 (1.4) | 2.6 [1.2 - 5.3] | 1.02E-02 |
