# Supplementary material for: Phenome-Wide Association Studies on a Quantitative Trait: Application to TPMT Enzyme Activity and Thiopurine Therapy in Pharmacogenomics
Source: PLoS Comput Biol. 2013 Dec 26;9(12):e1003405. doi: 10.1371/journal.pcbi.1003405 (PMC3873228; doi:10.1371/journal.pcbi.1003405)
Supplement: Table S4 — Distribution of PheWAS Codes from ICD-10 based aggregation. (DOCX) [file pcbi.1003405.s010.docx]

| **Code** | **Name** | **Number of patients (%)**  **N = 442** |
| --- | --- | --- |
| K50-K52 | Noninfective enteritis and colitis | 303/442 (68.6) |
| Z40-Z54 | Persons encountering health services for specific procedures and health care | 199/442 (45) |
| K55-K63 | Other diseases of intestines | 111/442 (25.1) |
| Z80-Z99 | Persons with potential health hazards related to family and personal history and certain conditions influencing health status | 81/442 (18.3) |
| R50-R69 | General symptoms and signs | 56/442 (12.7) |
| Z00-Z13 | Persons encountering health services for examination and investigation | 54/442 (12.2) |
| I10-I15 | Hypertensive diseases | 52/442 (11.8) |
| R10-R19 | Symptoms and signs involving the digestive system and abdomen | 49/442 (11.1) |
| N17-N19 | Renal failure | 48/442 (10.9) |
| E40-E46 | Malnutrition | 48/442 (10.9) |
| K90-K93 | Other diseases of the digestive system | 47/442 (10.6) |
| D50-D53 | Nutritional anaemias | 41/442 (9.3) |
| M30-M36 | Systemic connective tissue disorders | 41/442 (9.3) |
| E70-E90 | Metabolic disorders | 35/442 (7.9) |
| D60-D64 | Aplastic and other anaemias | 34/442 (7.7) |
| J95-J99 | Other diseases of the respiratory system | 32/442 (7.2) |
| I70-I79 | Diseases of arteries, arterioles and capillaries | 30/442 (6.8) |
| T80-T88 | Complications of surgical and medical care, not elsewhere classified | 28/442 (6.3) |
| I30-I52 | Other forms of heart disease | 28/442 (6.3) |
| D10-D36 | Benign neoplasms | 27/442 (6.1) |
| K20-K31 | Diseases of oesophagus, stomach and duodenum | 26/442 (5.9) |
| Z70-Z76 | Persons encountering health services in other circumstances | 26/442 (5.9) |
| Y40-Y59 | Drugs, medicaments and biological substances causing adverse effects in therapeutic use | 25/442 (5.7) |
| K80-K87 | Disorders of gallbladder, biliary tract and pancreas | 25/442 (5.7) |
| R00-R09 | Symptoms and signs involving the circulatory and respiratory systems | 24/442 (5.4) |
| I80-I89 | Diseases of veins, lymphatic vessels and lymph nodes, not elsewhere classified | 24/442 (5.4) |
| C00-C97 | Malignant neoplasms | 23/442 (5.2) |
| A00-A09 | Intestinal infectious diseases | 23/442 (5.2) |
| J80-J84 | Other respiratory diseases principally affecting the interstitium | 21/442 (4.8) |
| J09-J18 | Influenza and pneumonia | 21/442 (4.8) |
| K65-K67 | Diseases of peritoneum | 21/442 (4.8) |
| I20-I25 | Ischaemic heart diseases | 20/442 (4.5) |
| J40-J47 | Chronic lower respiratory diseases | 20/442 (4.5) |
| A30-A49 | Other bacterial diseases | 20/442 (4.5) |
| N00-N08 | Glomerular diseases | 19/442 (4.3) |
| I26-I28 | Pulmonary heart disease and diseases of pulmonary circulation | 19/442 (4.3) |
| B95-B97 | Bacterial, viral and other infectious agents | 18/442 (4.1) |
| K70-K77 | Diseases of liver | 18/442 (4.1) |
| C00-C75 | Malignant neoplasms, stated or presumed to be primary (of specified sites) | 17/442 (3.8) |
| D65-D69 | Coagulation defects, purpura and other haemorrhagic conditions | 17/442 (3.8) |
| E10-E14 | Diabetes mellitus | 16/442 (3.6) |
| Z20-Z29 | Persons with potential health hazards related to communicable diseases | 16/442 (3.6) |
| M05-M14 | Inflammatory polyarthropathies | 16/442 (3.6) |
| F10-F19 | Mental and behavioural disorders due to psychoactive substance use | 16/442 (3.6) |
| E65-E68 | Obesity and other hyperalimentation | 15/442 (3.4) |
| R70-R79 | Abnormal findings on examination of blood, without diagnosis | 14/442 (3.2) |
| E00-E07 | Disorders of thyroid gland | 13/442 (2.9) |
| F30-F39 | Mood [affective] disorders | 12/442 (2.7) |
| F40-F48 | Neurotic, stress-related and somatoform disorders | 11/442 (2.5) |
| J90-J94 | Other diseases of pleura | 11/442 (2.5) |
| N10-N16 | Renal tubulo-interstitial diseases | 11/442 (2.5) |
| I60-I69 | Cerebrovascular diseases | 11/442 (2.5) |
| G40-G47 | Episodic and paroxysmal disorders | 10/442 (2.3) |
| D80-D89 | Certain disorders involving the immune mechanism | 10/442 (2.3) |
| M45-M49 | Spondylopathies | 10/442 (2.3) |
| J20-J22 | Other acute lower respiratory infections | 9/442 (2) |
| R90-R94 | Abnormal findings on diagnostic imaging and in function studies, without diagnosis | 9/442 (2) |
| E20-E35 | Disorders of other endocrine glands | 9/442 (2) |
| D70-D77 | Other diseases of blood and blood-forming organs | 9/442 (2) |
| M70-M79 | Other soft tissue disorders | 9/442 (2) |
| B25-B34 | Other viral diseases | 9/442 (2) |
| M80-M85 | Disorders of bone density and structure | 8/442 (1.8) |
| E50-E64 | Other nutritional deficiencies | 7/442 (1.6) |
| Z55-Z65 | Persons with potential health hazards related to socioeconomic and psychosocial circumstances | 7/442 (1.6) |
| T36-T50 | Poisoning by drugs, medicaments and biological substances | 7/442 (1.6) |
| N80-N98 | Noninflammatory disorders of female genital tract | 6/442 (1.4) |
| U80-U89 | Bacterial agents resistant to antibiotics | 6/442 (1.4) |
| G50-G59 | Nerve, nerve root and plexus disorders | 6/442 (1.4) |
| D37-D48 | Neoplasms of uncertain or unknown behaviour | 6/442 (1.4) |
| M20-M25 | Other joint disorders | 6/442 (1.4) |
| N40-N51 | Diseases of male genital organs | 6/442 (1.4) |
| R40-R46 | Symptoms and signs involving cognition, perception, emotional state and behaviour | 5/442 (1.1) |
| J60-J70 | Lung diseases due to external agents | 5/442 (1.1) |
| Y83-Y84 | Surgical and other medical procedures as the cause of abnormal reaction of the patient, or of later complication, without mention of misadventure at the time of the procedure | 5/442 (1.1) |
| H15-H22 | Disorders of sclera, cornea, iris and ciliary body | 5/442 (1.1) |
| L80-L99 | Other disorders of the skin and subcutaneous tissue | 5/442 (1.1) |
| B35-B49 | Mycoses | 5/442 (1.1) |
| C30-C39 | Malignant neoplasms of respiratory and intrathoracic organs | 5/442 (1.1) |
| C76-C80 | Malignant neoplasms of ill-defined, secondary and unspecified sites | 5/442 (1.1) |
| C81-C96 | Malignant neoplasms of lymphoid, haematopoietic and related tissue | 5/442 (1.1) |
| R25-R29 | Symptoms and signs involving the nervous and musculoskeletal systems | 5/442 (1.1) |
| M50-M54 | Other dorsopathies | 5/442 (1.1) |
| L00-L08 | Infections of the skin and subcutaneous tissue | 5/442 (1.1) |
| M60-M63 | Disorders of muscles | 4/442 (0.9) |
| C15-C26 | Malignant neoplasms of digestive organs | 4/442 (0.9) |
| G60-G64 | Polyneuropathies and other disorders of the peripheral nervous system | 4/442 (0.9) |
| K00-K14 | Diseases of oral cavity, salivary glands and jaws | 4/442 (0.9) |
| K40-K46 | Hernia | 4/442 (0.9) |
| T51-T65 | Toxic effects of substances chiefly nonmedicinal as to source | 4/442 (0.9) |
| L40-L45 | Papulosquamous disorders | 4/442 (0.9) |
| I95-I99 | Other and unspecified disorders of the circulatory system | 4/442 (0.9) |
| J30-J39 | Other diseases of upper respiratory tract | 4/442 (0.9) |
| N25-N29 | Other disorders of kidney and ureter | 4/442 (0.9) |
| N30-N39 | Other diseases of urinary system | 4/442 (0.9) |
| I05-I09 | Chronic rheumatic heart diseases | 3/442 (0.7) |
| R30-R39 | Symptoms and signs involving the urinary system | 3/442 (0.7) |
| C60-C63 | Malignant neoplasms of male genital organs | 3/442 (0.7) |
| S70-S79 | Injuries to the hip and thigh | 3/442 (0.7) |
| L60-L75 | Disorders of skin appendages | 3/442 (0.7) |
| A15-A19 | Tuberculosis | 3/442 (0.7) |
| A50-A64 | Infections with a predominantly sexual mode of transmission | 3/442 (0.7) |
| B00-B09 | Viral infections characterized by skin and mucous membrane lesions | 3/442 (0.7) |
| B15-B19 | Viral hepatitis | 3/442 (0.7) |
| H90-H95 | Other disorders of ear | 3/442 (0.7) |
| G30-G32 | Other degenerative diseases of the nervous system | 3/442 (0.7) |
| N20-N23 | Urolithiasis | 2/442 (0.5) |
| R20-R23 | Symptoms and signs involving the skin and subcutaneous tissue | 2/442 (0.5) |
| H80-H83 | Diseases of inner ear | 2/442 (0.5) |
| L20-L30 | Dermatitis and eczema | 2/442 (0.5) |
| S30-S39 | Injuries to the abdomen, lower back, lumbar spine and pelvis | 2/442 (0.5) |
| M91-M94 | Chondropathies | 2/442 (0.5) |
| N70-N77 | Inflammatory diseases of female pelvic organs | 2/442 (0.5) |
| R80-R82 | Abnormal findings on examination of urine, without diagnosis | 2/442 (0.5) |
| X60-X84 | Intentional self-harm | 2/442 (0.5) |
| Y90-Y98 | Supplementary factors related to causes of morbidity and mortality classified elsewhere | 2/442 (0.5) |
| C51-C58 | Malignant neoplasms of female genital organs | 2/442 (0.5) |
| G00-G09 | Inflammatory diseases of the central nervous system | 2/442 (0.5) |
| B20-B24 | Human immunodeficiency virus [HIV] disease | 2/442 (0.5) |
| O00-O08 | Pregnancy with abortive outcome | 2/442 (0.5) |
| Q20-Q28 | Congenital malformations of the circulatory system | 2/442 (0.5) |
| L50-L54 | Urticaria and erythema | 2/442 (0.5) |
| F00-F09 | Organic, including symptomatic, mental disorders | 2/442 (0.5) |
| K35-K38 | Diseases of appendix | 2/442 (0.5) |
| M00-M03 | Infectious arthropathies | 1/442 (0.2) |
| M95-M99 | Other disorders of the musculoskeletal system and connective tissue | 1/442 (0.2) |
| G10-G13 | Systemic atrophies primarily affecting the central nervous system | 1/442 (0.2) |
| G80-G83 | Cerebral palsy and other paralytic syndromes | 1/442 (0.2) |
| B50-B64 | Protozoal diseases | 1/442 (0.2) |
| S40-S49 | Injuries to the shoulder and upper arm | 1/442 (0.2) |
| M15-M19 | Arthrosis | 1/442 (0.2) |
| M86-M90 | Other osteopathies | 1/442 (0.2) |
| R47-R49 | Symptoms and signs involving speech and voice | 1/442 (0.2) |
| J00-J06 | Acute upper respiratory infections | 1/442 (0.2) |
| J85-J86 | Suppurative and necrotic conditions of lower respiratory tract | 1/442 (0.2) |
| Y10-Y34 | Event of undetermined intent | 1/442 (0.2) |
| C45-C49 | Malignant neoplasms of mesothelial and soft tissue | 1/442 (0.2) |
| C64-C68 | Malignant neoplasms of urinary tract | 1/442 (0.2) |
| I00-I02 | Acute rheumatic fever | 1/442 (0.2) |
| G20-G26 | Extrapyramidal and movement disorders | 1/442 (0.2) |
| G70-G73 | Diseases of myoneural junction and muscle | 1/442 (0.2) |
| G90-G99 | Other disorders of the nervous system | 1/442 (0.2) |
| H60-H62 | Diseases of external ear | 1/442 (0.2) |
| H65-H75 | Diseases of middle ear and mastoid | 1/442 (0.2) |
| A65-A69 | Other spirochaetal diseases | 1/442 (0.2) |
| A80-A89 | Viral infections of the central nervous system | 1/442 (0.2) |
| O10-O16 | Oedema, proteinuria and hypertensive disorders in pregnancy, childbirth and the puerperium | 1/442 (0.2) |
| Q30-Q34 | Congenital malformations of the respiratory system | 1/442 (0.2) |
| H25-H28 | Disorders of lens | 1/442 (0.2) |
| H46-H48 | Disorders of optic nerve and visual pathways | 1/442 (0.2) |
| H49-H52 | Disorders of ocular muscles, binocular movement, accommodation and refraction | 1/442 (0.2) |
| H53-H54 | Visual disturbances and blindness | 1/442 (0.2) |
| H55-H59 | Other disorders of eye and adnexa | 1/442 (0.2) |
| F20-F29 | Schizophrenia, schizotypal and delusional disorders | 1/442 (0.2) |
| F50-F59 | Behavioural syndromes associated with physiological disturbances and physical factors | 1/442 (0.2) |
| F90-F98 | Behavioural and emotional disorders with onset usually occurring in childhood and adolescence | 1/442 (0.2) |
| S00-S09 | Injuries to the head | 1/442 (0.2) |
| S60-S69 | Injuries to the wrist and hand | 1/442 (0.2) |
| T66-T78 | Other and unspecified effects of external causes | 1/442 (0.2) |
| Z30-Z39 | Persons encountering health services in circumstances related to reproduction | 1/442 (0.2) |
