# Supplementary material for: Phenome-Wide Association Studies on a Quantitative Trait: Application to TPMT Enzyme Activity and Thiopurine Therapy in Pharmacogenomics
Source: PLoS Comput Biol. 2013 Dec 26;9(12):e1003405. doi: 10.1371/journal.pcbi.1003405 (PMC3873228; doi:10.1371/journal.pcbi.1003405)
Supplement: Table S5 — Distribution of PheWAS Codes from ICD-9-CM mapping aggregation. (DOCX) [file pcbi.1003405.s011.docx]

| **Code** | **Name** | **Number of patients (%)**  **N = 442** |
| --- | --- | --- |
| 555 | Regional enteritis | 239/442 (54.1) |
| 556 | Ulcerative colitis | 105/442 (23.8) |
| 401 | hypertension | 46/442 (10.4) |
| 560 | Intestinal obstruction without mention of hernia | 45/442 (10.2) |
| 569 | Other disorders of intestine | 43/442 (9.7) |
| 280 | Iron deficiency anemias | 41/442 (9.3) |
| 789 | Other symptoms involving abdomen and pelvis | 41/442 (9.3) |
| 584 | Acute renal failure | 35/442 (7.9) |
| 780 | Altered mental status | 32/442 (7.2) |
| 285 | Other and unspecified anemias | 31/442 (7) |
| 263 | Other and unspecified protein-calorie malnutrition | 31/442 (7) |
| 585 | Chronic kidney disease (CKD) | 29/442 (6.6) |
| 446 | Polyarteritis nodosa and allied conditions | 28/442 (6.3) |
| 558 | Other and unspecified noninfectious gastroenteritis and colitis | 28/442 (6.3) |
| 518 | Other diseases of lung | 27/442 (6.1) |
| 578 | Gastrointestinal hemorrhage | 25/442 (5.7) |
| 260 | severe protein-calorie malnutrition | 22/442 (5) |
| 272 | Disorders of lipoid metabolism | 21/442 (4.8) |
| 566 | Abscess of anal and rectal regions | 21/442 (4.8) |
| 786 | Symptoms involving respiratory system and other chest symptoms | 21/442 (4.8) |
| 785 | Symptoms involving cardiovascular system | 20/442 (4.5) |
| 567 | Peritonitis and retroperitoneal infections | 20/442 (4.5) |
| 411 | ISCHEMIC HEART DISEASE | 19/442 (4.3) |
| 516 | Other alveolar and parietoalveolar pneumonopathy | 18/442 (4.1) |
| 41 | Bacterial infection in conditions classified elsewhere and of unspecified site | 18/442 (4.1) |
| 38 | Septicemia | 18/442 (4.1) |
| 427 | Paroxysmal tachycardia, unspecified | 17/442 (3.8) |
| 250 | Diabetes Mellitus | 16/442 (3.6) |
| 440 | Atherosclerosis | 15/442 (3.4) |
| 428 | Heart failure | 15/442 (3.4) |
| 583 | Nephritis and nephropathy, not specified as acute or chronic | 14/442 (3.2) |
| 451 | Phlebitis and thrombophlebitis | 14/442 (3.2) |
| 453 | Pulmonary embolus and DVT | 14/442 (3.2) |
| 579 | Intestinal malabsorption | 14/442 (3.2) |
| 790 | Nonspecific findings on examination of blood | 13/442 (2.9) |
| 447 | Other disorders of arteries and arterioles | 13/442 (2.9) |
| 287 | Purpura and other hemorrhagic conditions | 13/442 (2.9) |
| 787 | Symptoms involving digestive system | 13/442 (2.9) |
| 405 | Secondary hypertension | 12/442 (2.7) |
| 296 | Bipolar | 12/442 (2.7) |
| 565 | Anal fissure and fistula | 11/442 (2.5) |
| 530 | Diseases of esophagus | 11/442 (2.5) |
| 276 | Disorders of fluid, electrolyte, and acid-base balance | 11/442 (2.5) |
| 305 | Nondependent abuse of drugs | 11/442 (2.5) |
| 564 | Functional digestive disorders, not elsewhere classified | 11/442 (2.5) |
| 415 | pulmonary heart disease | 10/442 (2.3) |
| 710 | Diffuse diseases of connective tissue | 10/442 (2.3) |
| 482 | Other bacterial pneumonia | 10/442 (2.3) |
| 517 | Lung involvement in conditions classified elsewhere | 10/442 (2.3) |
| 8 | Intestinal infections due to other organisms | 10/442 (2.3) |
| 466 | Acute bronchitis and bronchiolitis | 9/442 (2) |
| 211 | Benign neoplasm of other parts of digestive system | 9/442 (2) |
| 793 | Nonspecific abnormal findings on radiological and other examination of body structure | 9/442 (2) |
| 573 | Other disorders of liver | 9/442 (2) |
| 300 | Anxiety, dissociative and somatoform disorders | 9/442 (2) |
| 9 | Ill-defined intestinal infections | 8/442 (1.8) |
| 531 | Peptic ulcers | 8/442 (1.8) |
| 496 | Chronic airway obstruction, not elsewhere classified | 8/442 (1.8) |
| 486 | Pneumonia, organism unspecified | 8/442 (1.8) |
| 493 | Asthma | 8/442 (1.8) |
| 571 | Chronic liver disease and cirrhosis | 7/442 (1.6) |
| 410 | myocardial infarction | 7/442 (1.6) |
| 599 | Other disorders of urethra and urinary tract | 7/442 (1.6) |
| 425 | Cardiomyopathy | 7/442 (1.6) |
| 244 | Acquired hypothyroidism | 7/442 (1.6) |
| 255 | Disorders of adrenal glands | 7/442 (1.6) |
| 288 | Diseases of white blood cells | 7/442 (1.6) |
| 535 | Gastritis and duodenitis | 7/442 (1.6) |
| 511 | Pleurisy | 7/442 (1.6) |
| 576 | Other disorders of biliary tract | 7/442 (1.6) |
| 714 | Rheumatoid arthritis and other inflammatory polyarthropathies | 6/442 (1.4) |
| 491 | Chronic bronchitis | 6/442 (1.4) |
| 799 | Other ill-defined and unknown causes of morbidity and mortality | 6/442 (1.4) |
| 783 | Symptoms concerning nutrition, metabolism, and development | 6/442 (1.4) |
| 284 | Aplastic anemia and other bone marrow failure syndromes | 6/442 (1.4) |
| 574 | Cholelithiasis | 6/442 (1.4) |
| 577 | Diseases of pancreas | 6/442 (1.4) |
| 433 | Occlusion and stenosis of precerebral arteries | 6/442 (1.4) |
| 733 | Other disorders of bone and cartilage | 6/442 (1.4) |
| 788 | Symptoms involving urinary system | 5/442 (1.1) |
| 268 | Vitamin D deficiency | 5/442 (1.1) |
| 54 | Herpes simplex | 5/442 (1.1) |
| 238 | Neoplasm of uncertain behavior of other and unspecified sites and tissues | 5/442 (1.1) |
| 995 | Anaphylactic shock and angioedema | 5/442 (1.1) |
| 135 | Sarcoidosis | 5/442 (1.1) |
| 278 | Other hyperalimentation | 5/442 (1.1) |
| 279 | Disorders involving the immune mechanism | 5/442 (1.1) |
| 275 | Disorders of mineral metabolism | 5/442 (1.1) |
| 593 | Other disorders of kidney and ureter | 5/442 (1.1) |
| 286 | Coagulation defects | 5/442 (1.1) |
| 720 | Ankylosing spondylitis and other inflammatory spondylopathies | 5/442 (1.1) |
| 424 | Other diseases of endocardium | 5/442 (1.1) |
| 601 | Prostatitis | 5/442 (1.1) |
| 79 | Viral and chlamydial infection in conditions classified elsewhere and of unspecified site | 5/442 (1.1) |
| 581 | Nephrotic syndrome | 4/442 (0.9) |
| 444 | Arterial embolism and thrombosis | 4/442 (0.9) |
| 562 | Diverticula of intestine | 4/442 (0.9) |
| 572 | Liver abscess and sequelae of chronic liver disease | 4/442 (0.9) |
| 782 | Symptoms involving skin and other integumentary tissue | 4/442 (0.9) |
| 695 | Erythematous conditions | 4/442 (0.9) |
| 345 | Epilepsy and recurrent seizures | 4/442 (0.9) |
| 429 | Ill-defined descriptions and complications of heart disease | 4/442 (0.9) |
| 357 | Inflammatory and toxic neuropathy | 4/442 (0.9) |
| 242 | Thyrotoxicosis with or without goiter | 4/442 (0.9) |
| 964 | Poisoning by agents primarily affecting blood constituents | 4/442 (0.9) |
| 112 | Candidiasis | 4/442 (0.9) |
| 162 | Malignant neoplasm of trachea, bronchus, and lung | 4/442 (0.9) |
| 197 | Secondary malignant neoplasm of respiratory and digestive systems | 4/442 (0.9) |
| 202 | Other malignant neoplasms of lymphoid and histiocytic tissue | 4/442 (0.9) |
| 550 | HERNIA OF ABDOMINOPELVIC CAVITY | 4/442 (0.9) |
| 680 | Carbuncle and furuncle | 4/442 (0.9) |
| 696 | Psoriasis and similar disorders | 4/442 (0.9) |
| 512 | Pneumothorax | 4/442 (0.9) |
| 441 | Aortic aneurysm and dissection | 4/442 (0.9) |
| 580 | Acute glomerulonephritis | 4/442 (0.9) |
| 394 | CHRONIC RHEUMATIC HEART DISEASE | 3/442 (0.7) |
| 591 | Hydronephrosis | 3/442 (0.7) |
| 507 | Pneumonitis due to solids and liquids | 3/442 (0.7) |
| 726 | Peripheral enthesopathies and allied syndromes | 3/442 (0.7) |
| 459 | Other disorders of circulatory system | 3/442 (0.7) |
| 277 | Other and unspecified disorders of metabolism | 3/442 (0.7) |
| 724 | Other and unspecified disorders of back | 3/442 (0.7) |
| 420 | pericarditis | 3/442 (0.7) |
| 422 | Acute myocarditis | 3/442 (0.7) |
| 185 | Malignant neoplasm of prostate | 3/442 (0.7) |
| 484 | Pneumonia in infectious diseases classified elsewhere | 3/442 (0.7) |
| 707 | Chronic ulcer of skin | 3/442 (0.7) |
| 963 | Poisoning by primarily systemic agents | 3/442 (0.7) |
| 70 | Viral hepatitis | 3/442 (0.7) |
| 78 | Other diseases due to viruses and Chlamydiae | 3/442 (0.7) |
| 117 | Other mycoses | 3/442 (0.7) |
| 478 | Other diseases of upper respiratory tract | 3/442 (0.7) |
| 10 | TUBERCULOSIS | 3/442 (0.7) |
| 304 | Drug dependence | 3/442 (0.7) |
| 389 | Hearing loss | 3/442 (0.7) |
| 426 | Conduction disorders | 3/442 (0.7) |
| 423 | Other diseases of pericardium | 3/442 (0.7) |
| 492 | Emphysema | 3/442 (0.7) |
| 729 | Other disorders of soft tissues | 3/442 (0.7) |
| 590 | Infections of kidney | 3/442 (0.7) |
| 711 | Arthropathy associated with infections | 3/442 (0.7) |
| 289 | Other diseases of blood and blood-forming organs | 3/442 (0.7) |
| 413 | Angina pectoris | 2/442 (0.5) |
| 557 | Vascular insufficiency of intestine | 2/442 (0.5) |
| 575 | Other disorders of gallbladder | 2/442 (0.5) |
| 537 | Other disorders of stomach and duodenum | 2/442 (0.5) |
| 435 | Transient cerebral ischemia | 2/442 (0.5) |
| 455 | Hemorrhoids | 2/442 (0.5) |
| 910 | superficial, contusion, crushing injuries | 2/442 (0.5) |
| 693 | Dermatitis due to substances taken internally | 2/442 (0.5) |
| 153 | Malignant neoplasm of colon | 2/442 (0.5) |
| 386 | Vertiginous syndromes and other disorders of vestibular system | 2/442 (0.5) |
| 784 | Symptoms involving head and neck | 2/442 (0.5) |
| 252 | Disorders of parathyroid gland | 2/442 (0.5) |
| 442 | Other aneurysm | 2/442 (0.5) |
| 269 | Other nutritional deficiencies | 2/442 (0.5) |
| 35 | Erysipelas | 2/442 (0.5) |
| 437 | Other and ill-defined cerebrovascular disease | 2/442 (0.5) |
| 622 | Noninflammatory disorders of cervix | 2/442 (0.5) |
| 458 | Hypotension | 2/442 (0.5) |
| 454 | Varicose veins | 2/442 (0.5) |
| 457 | Noninfectious disorders of lymphatic channels | 2/442 (0.5) |
| 303 | Alcohol dependence syndrome | 2/442 (0.5) |
| 291 | Substance-induced mental disorders | 2/442 (0.5) |
| 331 | Other cerebral degenerations | 2/442 (0.5) |
| 42 | Human immunodeficiency virus [HIV] disease | 2/442 (0.5) |
| 540 | Appendicitis | 2/442 (0.5) |
| 614 | Inflammatory disease of ovary, fallopian tube, pelvic cellular tissue, and peritoneum | 2/442 (0.5) |
| 355 | Mononeuritis of lower limb | 2/442 (0.5) |
| 198 | Secondary malignant neoplasm of other specified sites | 2/442 (0.5) |
| 379 | Other disorders of eye | 2/442 (0.5) |
| 370 | Keratitis | 2/442 (0.5) |
| 704 | Diseases of hair and hair follicles | 2/442 (0.5) |
| 481 | PNEUMOCOCCAL PNEUMONIA | 2/442 (0.5) |
| 791 | Nonspecific findings on examination of urine | 2/442 (0.5) |
| 582 | Chronic glomerulonephritis | 1/442 (0.2) |
| 713 | Arthropathy associated with other disorders classified elsewhere | 1/442 (0.2) |
| 436 | Acute, but ill-defined cerebrovascular disease | 1/442 (0.2) |
| 274 | Gout | 1/442 (0.2) |
| 618 | Genital prolapse | 1/442 (0.2) |
| 617 | Endometriosis | 1/442 (0.2) |
| 840 | Sprains and strains | 1/442 (0.2) |
| 342 | Hemiplegia and hemiparesis | 1/442 (0.2) |
| 681 | Superficial cellulitis and abscess | 1/442 (0.2) |
| 293 | Transient mental disorders due to conditions classified elsewhere | 1/442 (0.2) |
| 136 | Other and unspecified infectious and parasitic diseases | 1/442 (0.2) |
| 335 | Anterior horn cell disease | 1/442 (0.2) |
| 796 | Other nonspecific abnormal findings | 1/442 (0.2) |
| 570 | Acute and subacute necrosis of liver | 1/442 (0.2) |
| 781 | Symptoms involving nervous and musculoskeletal systems | 1/442 (0.2) |
| 586 | RENAL FAILURE NOS | 1/442 (0.2) |
| 588 | Disorders resulting from impaired renal function | 1/442 (0.2) |
| 443 | Other peripheral vascular disease | 1/442 (0.2) |
| 513 | Abscess of lung and mediastinum | 1/442 (0.2) |
| 519 | Other diseases of respiratory system | 1/442 (0.2) |
| 465 | Acute upper respiratory infections of multiple or unspecified sites | 1/442 (0.2) |
| 715 | Osteoarthrosis and allied disorders | 1/442 (0.2) |
| 712 | Crystal arthropathies | 1/442 (0.2) |
| 596 | Other disorders of bladder | 1/442 (0.2) |
| 505 | PNEUMOCONIOSIS NOS | 1/442 (0.2) |
| 508 | Respiratory conditions due to other and unspecified external agents | 1/442 (0.2) |
| 273 | Disorders of plasma protein metabolism | 1/442 (0.2) |
| 431 | INTRACEREBRAL HEMORRHAGE | 1/442 (0.2) |
| 434 | Occlusion of cerebral arteries | 1/442 (0.2) |
| 438 | Late effects of cerebrovascular disease | 1/442 (0.2) |
| 619 | Fistula involving female genital tract | 1/442 (0.2) |
| 621 | Disorders of uterus, not elsewhere classified | 1/442 (0.2) |
| 626 | Disorders of menstruation and other abnormal bleeding from female genital tract | 1/442 (0.2) |
| 627 | Menopausal and postmenopausal disorders | 1/442 (0.2) |
| 728 | Disorders of muscle, ligament, and fascia | 1/442 (0.2) |
| 494 | Bronchiectasis | 1/442 (0.2) |
| 215 | Other benign neoplasm of connective and other soft tissue | 1/442 (0.2) |
| 220 | Benign neoplasm of ovary | 1/442 (0.2) |
| 223 | Benign neoplasm of kidney and other urinary organs | 1/442 (0.2) |
| 228 | Hemangioma and lymphangioma, any site | 1/442 (0.2) |
| 217 | Benign neoplasm of breast | 1/442 (0.2) |
| 212 | Benign neoplasm of respiratory and intrathoracic organs | 1/442 (0.2) |
| 723 | Other disorders of cervical region | 1/442 (0.2) |
| 452 | Portal vein thrombosis | 1/442 (0.2) |
| 158 | Malignant neoplasm of retroperitoneum and peritoneum | 1/442 (0.2) |
| 154 | Malignant neoplasm of rectum, rectosigmoid junction, and anus | 1/442 (0.2) |
| 421 | endocarditis | 1/442 (0.2) |
| 383 | Mastoiditis and related conditions | 1/442 (0.2) |
| 380 | Disorders of external ear | 1/442 (0.2) |
| 312 | Conduct disorders | 1/442 (0.2) |
| 307 | Eating disorders | 1/442 (0.2) |
| 308 | Acute reaction to stress | 1/442 (0.2) |
| 339 | Other headache syndromes | 1/442 (0.2) |
| 290 | Dementias | 1/442 (0.2) |
| 295 | Schizophrenic disorders | 1/442 (0.2) |
| 188 | Malignant neoplasm of bladder | 1/442 (0.2) |
| 180 | Malignant neoplasm of cervix uteri | 1/442 (0.2) |
| 184 | Malignant neoplasm of other and unspecified female genital organs | 1/442 (0.2) |
| 333 | Other extrapyramidal disease and abnormal movement disorders | 1/442 (0.2) |
| 330 | Cerebral degenerations usually manifest in childhood | 1/442 (0.2) |
| 332 | Parkinson's disease | 1/442 (0.2) |
| 336 | Other diseases of spinal cord | 1/442 (0.2) |
| 600 | Hyperplasia of prostate | 1/442 (0.2) |
| 49 | Other non-arthropod-borne viral diseases of central nervous system | 1/442 (0.2) |
| 91 | Syphilis | 1/442 (0.2) |
| 101 | Vincent's angina | 1/442 (0.2) |
| 174 | Malignant neoplasm of female breast | 1/442 (0.2) |
| 391 | Rheumatic fever with heart involvement | 1/442 (0.2) |
| 642 | Hypertension complicating pregnancy, childbirth, and the puerperium | 1/442 (0.2) |
| 940 | BURNS | 1/442 (0.2) |
| 955 | Injury to peripheral nerve(s) of shoulder girdle and upper limb | 1/442 (0.2) |
| 325 | Phlebitis and thrombophlebitis of intracranial venous sinuses | 1/442 (0.2) |
| 321 | Meningitis due to other organisms | 1/442 (0.2) |
| 352 | Disorders of other cranial nerves | 1/442 (0.2) |
| 359 | Muscular dystrophies and other myopathies | 1/442 (0.2) |
| 353 | Nerve root and plexus disorders | 1/442 (0.2) |
| 350 | Trigeminal nerve disorders | 1/442 (0.2) |
| 354 | Mononeuritis of upper limb and mononeuritis multiplex | 1/442 (0.2) |
| 745 | Bulbus cordis anomalies and anomalies of cardiac septal closure | 1/442 (0.2) |
| 748 | Anomalies of respiratory system, congenital | 1/442 (0.2) |
| 746 | Other congenital anomalies of heart | 1/442 (0.2) |
| 747 | Other congenital anomalies of circulatory system | 1/442 (0.2) |
| 476 | Chronic laryngitis and laryngotracheitis | 1/442 (0.2) |
| 473 | Chronic sinusitis | 1/442 (0.2) |
| 526 | Diseases of the jaws | 1/442 (0.2) |
| 523 | Gingival and periodontal diseases | 1/442 (0.2) |
| 525 | Other diseases and conditions of the teeth and supporting structures | 1/442 (0.2) |
| 528 | Diseases of the oral soft tissues, excluding lesions specific for gingiva and tongue | 1/442 (0.2) |
| 161 | Malignant neoplasm of larynx | 1/442 (0.2) |
| 196 | Secondary and unspecified malignant neoplasm of lymph nodes | 1/442 (0.2) |
| 203 | Multiple myeloma and immunoproliferative neoplasms | 1/442 (0.2) |
| 6 | Amebiasis | 1/442 (0.2) |
| 5 | Other food poisoning (bacterial) | 1/442 (0.2) |
| 75 | INFECTIOUS MONONUCLEOSIS | 1/442 (0.2) |
| 53 | Herpes zoster | 1/442 (0.2) |
| 235 | Neoplasm of uncertain behavior of digestive and respiratory systems | 1/442 (0.2) |
| 366 | Cataract | 1/442 (0.2) |
| 364 | Disorders of iris and ciliary body | 1/442 (0.2) |
| 378 | Strabismus and other disorders of binocular eye movements | 1/442 (0.2) |
| 377 | Disorders of optic nerve and visual pathways | 1/442 (0.2) |
| 369 | Blindness and low vision | 1/442 (0.2) |
| 245 | Thyroiditis | 1/442 (0.2) |
| 241 | Nontoxic nodular goiter | 1/442 (0.2) |
| 969 | Poisoning by psychotropic agents | 1/442 (0.2) |
| 962 | Poisoning by hormones and synthetic substitutes | 1/442 (0.2) |
| 965 | Poisoning by analgesics, antipyretics, and antirheumatics | 1/442 (0.2) |
| 701 | Other hypertrophic and atrophic conditions of skin | 1/442 (0.2) |
| 706 | Diseases of sebaceous glands | 1/442 (0.2) |
| 709 | Other disorders of skin and subcutaneous tissue | 1/442 (0.2) |
| 487 | Influenza | 1/442 (0.2) |
| 568 | Other disorders of peritoneum | 1/442 (0.2) |
