# Supplementary material for: Phenome-Wide Association Studies on a Quantitative Trait: Application to TPMT Enzyme Activity and Thiopurine Therapy in Pharmacogenomics
Source: PLoS Comput Biol. 2013 Dec 26;9(12):e1003405. doi: 10.1371/journal.pcbi.1003405 (PMC3873228; doi:10.1371/journal.pcbi.1003405)
Supplement: Table S6 — Results of the Phenome-wide association study (PheWAS) between very high TPMT activity patients and other TPMT activity patients for the ICD-9-CM mapping aggregation. The ICD-9-CM mapping aggregation corresponds to 771 groups of codes. Associations are assessed using logistic regression. Only PheWAS codes with a p-value<0.05 are reported here. The q value for false discovery rate (FDR) was q = 0.2. The p-value must be under the calculated FDR threshold to be considered as significant. TPMTa: thiopurine S-methyltransferase activity. Low TPMTa: <8.5 nmol/h/mL red blood cells; Very high TPMTa: ≥15.0 nmol/h/mL red blood cells; Normal TPMTa: in between. (DOCX) [file pcbi.1003405.s012.docx]

| **Name** | **vhTPMTa**  **n = 76** | **Other TPMTa**  **n = 366** | **Odds-ratio [95%CI]** | **p-value** | **FDR threshold** |
| --- | --- | --- | --- | --- | --- |
| **Iron deficiency anemia** | **15/57(26.3)** | **26/289(9)** | **3.6 [1.8-7.4]** | **0.0004** | **0.0008** |
| **Diabetes Mellitus** | **8/70(11.4)** | **8/328(2.4)** | **5.2 [1.9-14.3]** | **0.00156** | **0.00161** |
| Secondary hypertension | 6/57(10.5) | 6/286(2.1) | 5.5 [1.7-17.7] | 0.0043 | 0.0024 |
| Other and unspecified anemias | 10/52(19.2) | 21/284(7.4) | 3 [1.3-6.8] | 0.0091 | 0.0032 |
| Gastrointestinal hemorrhage | 8/55(14.5) | 17/289(5.9) | 2.7 [1.1-6.7] | 0.0283 | 0.004 |
| Acute bronchitis and bronchiolitis | 4/63(6.3) | 5/322(1.6) | 4.3 [1.1-16.5] | 0.0334 | 0.0048 |
| Acute renal failure | 10/60(16.7) | 25/308(8.1) | 2.3 [1-5] | 0.0433 | 0.0056 |
