# Supplementary material for: Phenome-Wide Association Studies on a Quantitative Trait: Application to TPMT Enzyme Activity and Thiopurine Therapy in Pharmacogenomics
Source: PLoS Comput Biol. 2013 Dec 26;9(12):e1003405. doi: 10.1371/journal.pcbi.1003405 (PMC3873228; doi:10.1371/journal.pcbi.1003405)
Supplement: Table S7 — Results of the Phenome-wide association study (PheWAS) between very high TPMT activity patients and other TPMT activity patients for the ICD-10 based aggregation. The ICD-10 based aggregation corresponds to 256 groups of codes. Only PheWAS codes with a p-value<0.05 are reported here. Associations are assessed using logistic regression. The q value for false discovery rate (FDR) was q = 0.2. The p-value must be under the calculated FDR threshold to be considered as significant. TPMTa: thiopurine S-methyltransferase activity. Low TPMTa: <8.5 nmol/h/mL red blood cells; Very high TPMTa: ≥15.0 nmol/h/mL red blood cells; Normal TPMTa: in between. (DOCX) [file pcbi.1003405.s013.docx]

| **Name** | **vhTPMTa**  **n = 76** | **Other TPMTa**  **n = 366** | **Odds-ratio [95%CI]** | **p-value** | **FDR threshold** |
| --- | --- | --- | --- | --- | --- |
| **Nutritional anemia** | **15/57(26.3)** | **26/284(9.2)** | **3.5 [1.7-7.2]** | **0.0005** | **0.0014** |
| **Diabetes mellitus** | **8/51(15.7)** | **8/254(3.1)** | **5.7 [2-16.1]** | **0.00093** | **0.00289** |
| Aplastic and other anemias | 10/52(19.2) | 24/282(8.5) | 2.6 [1.1-5.7] | 0.0224 | 0.0043 |
| Other acute lower respiratory infections | 4/54(7.4) | 5/278(1.8) | 4.4 [1.1-16.8] | 0.0322 | 0.0058 |
