# Supplementary material for: Phenome-Wide Association Studies on a Quantitative Trait: Application to TPMT Enzyme Activity and Thiopurine Therapy in Pharmacogenomics
Source: PLoS Comput Biol. 2013 Dec 26;9(12):e1003405. doi: 10.1371/journal.pcbi.1003405 (PMC3873228; doi:10.1371/journal.pcbi.1003405)
Supplement: Table S8 — Results of the Phenome-wide association study (PheWAS) between low TPMT activity patients and other TPMT activity patients for the ICD-9-CM mapping aggregation. The ICD-9-CM mapping aggregation corresponds to 771 groups of codes. Only PheWAS codes with a p-value<0.05 are reported here. Associations are assessed using logistic regression. The q value for false discovery rate (FDR) was q = 0.2. The p-value must be under the calculated FDR threshold to be considered as significant. TPMTa: thiopurine S-methyltransferase activity. Low TPMTa: <8.5 nmol/h/mL red blood cells; Very high TPMTa: ≥15.0 nmol/h/mL red blood cells; Normal TPMTa: in between. (DOCX) [file pcbi.1003405.s014.docx]

| **Name** | **lowTPMTa**  **n = 42** | **Other TPMTa**  **n = 400** | **Odds-ratio [95%CI]** | **p-value** | **FDR threshold** |
| --- | --- | --- | --- | --- | --- |
| Symptoms involving digestive system | 4/32(12.5) | 9/271(3.3) | 4.2 [1.2-14.4] | 0.02 | 0.0008 |
| Nonspecific abnormal findings on radiological and other examination of body structure | 3/38(7.9) | 6/332(1.8) | 4.7 [1.1-19.4] | 0.03 | 0.0016 |
| Other disorders of kidney and ureter | 2/37(5.4) | 3/334(0.9) | 6.3 [1-39] | 0.04 | 0.002 |
