# Supplementary material for: Phenome-Wide Association Studies on a Quantitative Trait: Application to TPMT Enzyme Activity and Thiopurine Therapy in Pharmacogenomics
Source: PLoS Comput Biol. 2013 Dec 26;9(12):e1003405. doi: 10.1371/journal.pcbi.1003405 (PMC3873228; doi:10.1371/journal.pcbi.1003405)
Supplement: Table S9 — Results of the Phenome-wide association study (PheWAS) between low TPMT activity patients and other TPMT activity patients for the ICD10 based aggregation. The ICD-10 based aggregation corresponds to 256 groups of codes. Only PheWAS codes with a p-value<0.05 are reported here. Associations are assessed using logistic regression. The q value for false discovery rate (FDR) was q = 0.2. The p-value must be under the calculated FDR threshold to be considered as significant. (DOCX) [file pcbi.1003405.s015.docx]

| **Name** | **lowTPMTa**  **n = 42** | **Other TPMTa**  **n = 400** | **Odds-ratio [95%CI]** | **p-value** | **FDR threshold** |
| --- | --- | --- | --- | --- | --- |
| Mycoses | 3/34(8.8) | 2/292(0.7) | 14 [2.3-87.2] | 0.0046 | 0.001 |
| Persons with potential health hazards related to socioeconomic and psychosocial circumstances | 3/17(17.6) | 4/137(2.9) | 7.1 [1.4-35.1] | 0.0158 | 0.002 |
| Abnormal findings on diagnostic imaging and in function studies, without diagnosis | 3/29(10.3) | 6/259(2.3) | 4.9 [1.1-20.6] | 0.0317 | 0.004 |
| Infections of the skin and subcutaneous tissue | 2/36(5.6) | 3/333(0.9) | 6.5 [1-40.1] | 0.0448 | 0.005 |
| Spondylopathy | 3/32(9.4) | 7/287(2.4) | 4.1 [1-16.9] | 0.0477 | 0.007 |
