# Supplementary material for: Phenome-Wide Association Studies on a Quantitative Trait: Application to TPMT Enzyme Activity and Thiopurine Therapy in Pharmacogenomics
Source: PLoS Comput Biol. 2013 Dec 26;9(12):e1003405. doi: 10.1371/journal.pcbi.1003405 (PMC3873228; doi:10.1371/journal.pcbi.1003405)
Supplement: Table S10 — Results of the low-value case biological test analyses between low TPMT activity patients and other patients with normal and very high TPMT activity. Global approach: a low-value case is defined as at least one occurrence, over the study period, of biological test result below the low threshold defined in Table 1. Frequency-based approach: for a given patient, the frequency of low-value encounters is defined as the number of encounters with at least one occurrence below the low threshold divided by the number of encounters (mean low-value encounter frequencies are reported). Low-value case analyses have not been performed on alanine aminotransferase, aspartate aminotransferase and gamma glutamyl-transpeptidase test results, as a low threshold is not relevant for these tests. (DOCX) [file pcbi.1003405.s016.docx]

| **Biological tests** | **Global approach** | | | | **Frequency-based approach** | | |
| --- | --- | --- | --- | --- | --- | --- | --- |
|  | **lowTPMTa**  **n = 42**  **(%)** | **nTPMTa + vhTPTMa**  **n = 400**  **(%)** | **Odds Ratio**  **[95%CI]** | **p value** | **vhTPMTa**  **encounter**  **frequency** | **nTPMTa + lowTPTMa**  **encounter**  **frequency** | **p value** |
| **Leukocyte count** | 6/42(14.3) | 98/394(24.9) | 0.5 [0.2-1.3] | 0.2 | **0.037** | **0.104** | **0.002** |
| **Neutrophil count** | 3/41(7.3) | 41/392(10.5) | 0.7 [0.1-2.3] | 0.8 | **0.009** | **0.027** | **0.013** |
| Red blood cell count | 32/42(76.2) | 340/394(86.3) | 0.5 [0.2-1.2] | 0.1 | 0.63 | 0.732 | 0.151 |
| Hemoglobin | 9/42(21.4) | 116/394(29.4) | 0.7 [0.3-1.5] | 0.4 | 0.076 | 0.109 | 0.356 |
| Mean corpuscular volume | 16/42(38.1) | 186/394(47.2) | 0.7 [0.3-1.4] | 0.3 | 0.299 | 0.33 | 0.661 |
| Platelet count | 3/42(7.1) | 24/394(6.1) | 1.2 [0.2-4.2] | 0.7 | 0.032 | 0.022 | 0.706 |
| Glycemia | 31/35(88.6) | 301/327(92) | 0.7 [0.2-2.8] | 0.5 | 0.738 | 0.763 | 0.674 |
| Alkaline phosphatase | 13/39(33.3) | 129/368(35.1) | 0.9 [0.4-1.9] | 1 | 0.184 | 0.184 | 0.991 |
