# Supplementary material for: Phenome-Wide Association Studies on a Quantitative Trait: Application to TPMT Enzyme Activity and Thiopurine Therapy in Pharmacogenomics
Source: PLoS Comput Biol. 2013 Dec 26;9(12):e1003405. doi: 10.1371/journal.pcbi.1003405 (PMC3873228; doi:10.1371/journal.pcbi.1003405)
Supplement: Table S11 — Results of the high-value-case biological test analyses between low TPMT activity (lowTPMTa) patients and other patients. Global approach: a high-value case is defined as at least one occurrence, over the study period, of biological test result above the high threshold defined in Table 1. Frequency-based approach: for a given patient, the frequency of low-value encounters is defined as the number of encounters with at least one occurrence below the low threshold divided by the number of encounters (mean low-value encounter frequencies are reported). TPMTa: thiopurine S-methyltransferase activity. lowTPMTa: low TPMTa (<8.5 nmol/h/mL red blood cells); vhTPMTa: very high TPMTa (≥15.0 nmol/h/mL red blood cells); nTPMTa: normal TPMTa (in between). (DOCX) [file pcbi.1003405.s017.docx]

| **Biological test** | **Global approach *** | | | | **Frequency-based approach **** | | |
| --- | --- | --- | --- | --- | --- | --- | --- |
|  | **lowTPMTa**  **n = 42**  **(%)** | **nTPMTa + vhTPTMa**  **n = 400**  **(%)** | **Odds Ratio**  **[IC95]** | **p value** | **vhTPMTa**  **encounter**  **frequency** | **nTPMTa + lowTPTMa**  **encounter**  **frequency** | **p value** |
| Leukocyte count | 33/42(78.6) | 258/394(65.5) | 1.9 [0.9-4.7] | 0.1 | 0.479 | 0.368 | 0.059 |
| Neutrophil count | 24/41(58.5) | 188/392(48) | 1.5 [0.8-3.1] | 0.3 | 0.23 | 0.207 | 0.628 |
| Red blood cell count | 2/42(4.8) | 26/394(6.6) | 0.7 [0.1-3] | 1 | 0.01 | 0.02 | 0.267 |
| Hemoglobin | 3/42(7.1) | 26/394(6.6) | 1.1 [0.2-3.8] | 0.8 | 0.009 | 0.018 | 0.205 |
| Mean corpuscular volume | 14/42(33.3) | 148/394(37.6) | 0.8 [0.4-1.7] | 0.6 | 0.206 | 0.227 | 0.702 |
| Platelet count | 19/42(45.2) | 171/394(43.4) | 1.1 [0.5-2.1] | 0.9 | 0.178 | 0.215 | 0.447 |
| **Glycemia** | 1/34(2.9) | 24/311(7.7) | 0.4 [0-2.4] | 0.5 | **0.01** | **0.041** | **0.003** |
| Alkaline phosphatase | 21/39(53.8) | 166/368(45.1) | 1.4 [0.7-2.9] | 0.3 | 0.344 | 0.282 | 0.351 |
| Alanine aminotransferase | 12/41(29.3) | 98/373(26.3) | 1.2 [0.5-2.5] | 0.7 | 0.152 | 0.099 | 0.269 |
| Aspartate aminotransferase | 8/41(19.5) | 63/373(16.9) | 1.2 [0.5-2.8] | 0.7 | 0.076 | 0.058 | 0.582 |
| Gamma glutamyl-transpeptidase | 11/38(28.9) | 89/369(24.1) | 1.3 [0.6-2.8] | 0.6 | 0.165 | 0.127 | 0.476 |
